# Peripheral T Cell Subpopulations as a Potential Surrogate Biomarker during Atezolizumab plus Bevacizumab Treatment for Hepatocellular Carcinoma

**DOI:** 10.3390/cancers16071328

**Published:** 2024-03-28

**Authors:** Yuki Shirane, Yasutoshi Fujii, Atsushi Ono, Hikaru Nakahara, Clair Nelson Hayes, Ryoichi Miura, Serami Murakami, Naoya Sakamoto, Shinsuke Uchikawa, Hatsue Fujino, Takashi Nakahara, Eisuke Murakami, Masami Yamauchi, Daiki Miki, Tomokazu Kawaoka, Koji Arihiro, Masataka Tsuge, Shiro Oka

**Affiliations:** 1 Department of Gastroenterology, Graduate School of Biomedical and Health Sciences, Hiroshima University, Hiroshima 734-8551, Japan; yuki0415@hiroshima-u.ac.jp (Y.S.); fujiiyasu@hiroshima-u.ac.jp (Y.F.); hnkhr@hiroshima-u.ac.jp (H.N.); nelsonhayes@hiroshima-u.ac.jp (C.N.H.); ryoichim@hiroshima-u.ac.jp (R.M.); serami@hiroshima-u.ac.jp (S.M.); shinuchi@hiroshima-u.ac.jp (S.U.); fujino920@hiroshima-u.ac.jp (H.F.); nakahara@hiroshima-u.ac.jp (T.N.); emusuke@hiroshima-u.ac.jp (E.M.); daikimiki@hiroshima-u.ac.jp (D.M.); kawaokatomo@hiroshima-u.ac.jp (T.K.); tsuge@hiroshima-u.ac.jp (M.T.); oka4683@hiroshima-u.ac.jp (S.O.); 2 Department of Clinical Oncology, Graduate School of Biomedical and Health Sciences, Hiroshima University, Hiroshima 734-8551, Japan; myamauchi@hiroshima-u.ac.jp; 3 Division of Pathology, Exploratory Oncology Research & Clinical Trial Center, National Cancer Center, Kashiwa, Chiba 277-8577, Japan; naosakam@east.ncc.go.jp; 4 Department of Anatomical Pathology, Hiroshima University Hospital, Hiroshima 734-8551, Japan; arihiro@hiroshima-u.ac.jp

**Keywords:** hepatocellular carcinoma, peripheral T cell subpopulation, central memory T cell, effector memory T cell, atezolizumab plus bevacizumab

## Abstract

**Simple Summary:**

In this study, we evaluated the status and dynamics of peripheral T cell subpopulations in hepatocellular carcinoma (HCC) patients receiving immune checkpoint blockade with atezolizumab plus bevacizumab (Atez/Bev) treatment and explored biomarkers that are predictive of therapeutic response. The results showed that a higher proportion of CD8+ central memory T cells at baseline is associated with tumor inflammation in HCC, as well as with longer progression-free survival in response to Atez/Bev treatment, especially in cases with an increased proportion of CD8+ effector memory T cells after 3 weeks. Our findings suggest that the peripheral T cell subpopulation can serve as a potential noninvasive biomarker predicting benefits from Atez/Bev treatment.

**Abstract:**

The therapeutic benefits of the immunotherapeutic combination of atezolizumab and bevacizumab (Atez/Bev) in hepatocellular carcinoma (HCC) vary. Therapeutic biomarkers might help improve outcomes for HCC patients receiving Atez/Bev therapy. The role of systemic immune profiles in HCC progression also remains unclear. This study aimed to evaluate the status and dynamics of peripheral T cell subpopulations in HCC patients receiving Atez/Bev treatment and to explore biomarkers predictive of a therapeutic response. We enrolled 83 unresectable advanced HCC patients who commenced Atez/Bev treatment at our hospital between October 2020 and June 2022. Peripheral T cell subpopulations in peripheral blood mononuclear cells at baseline and 3 weeks post-treatment were investigated using flow cytometry and compared with those in control samples from 18 healthy individuals. We retrospectively analyzed the association between peripheral T cell subpopulation profiles and clinical outcomes. Baseline peripheral T cell subpopulations could be profiled in 70 patients with sufficient cell counts, among whom 3-week subpopulations could be evaluated in 51 patients. Multivariate analysis showed that a high baseline proportion of CD8+ central memory T (TCM) cells was independently associated with longer progression-free survival (PFS). Further, overall survival (OS) was significantly prolonged in patients with increased CD8+ effector memory T (TEM) cell proportions. In conclusion, TCM proportion at baseline might be a good indicator of the efficacy of Atez/Bev therapy. Furthermore, observation of increasing TEM proportions might be an early predictor of the potential clinical benefits of treatment.

## 1. Introduction

Hepatocellular carcinoma (HCC) is one of the most common malignant tumors and is a leading cause of cancer-related deaths globally [1]. Recently, atezolizumab plus bevacizumab (Atez/Bev) combination treatment, the first HCC-targeted immunotherapy, was approved as a first-line treatment for HCC, based on an international, open-label, phase 3 trial (IMbrave150; NCT03434379) [2]. However, its therapeutic benefits vary by case, and only a fraction of patients show a durable response. Another important clinical challenge is the identification of patients who are likely to respond to immune checkpoint inhibitor (ICI) treatment. Relevant biomarkers of the likely outcomes might help to identify patients who are most likely to benefit from Atez/Bev treatment, helping to guide treatment decisions and improve patient outcomes. One potential biomarker is programmed death-ligand 1 (PD-L1), a protein that is expressed on the surface of tumor cells and binds to the PD-1 receptor on T cells, inhibiting the immune response. Zhu et al. reported that messenger RNA expression of CD274 (PD-L1) in HCC tissue is higher in patients with a complete response/partial response (CR/PR) than in those with stable disease/progressive disease (SD/PD), and that high expression of CD274 (defined by a median split) is also associated with longer progression-free survival (PFS) than low expression in HCC patients treated with Atez/Bev [2]. Regarding blood-based non-invasive biomarkers, several candidate markers, such as IL-6 [3,4], neutrophil-lymphocyte ratio (NLR) [5,6], plasma growth hormone [7], and CRAFITY (CRP and AFP in ImmunoTherapY) score [8] have been documented as non-invasive prognostic or predictive biomarkers in Atez/Bev therapy. However, no consensus biomarker has yet been established.

Immunotherapy targets the immune system or the tumor microenvironment (TME) of the patient in order to induce an antitumor immune response. Although numerous factors play a role in cancer immunity, T cells play a major role. Based on their ability to expand clonally and exert cytotoxic effects, the effector T (Teff) cells are recognized as important mediators of the anti-tumor response. In a previous study on HCC patients treated with Atez/Bev, higher expression of Teff signature genes (CXCL9, PRF1, and GZMB) was reportedly associated with longer PFS in the molecular analysis of 90 baseline tumors, and a high regulatory T cell (Treg) to Teff ratio was associated with reduced clinical benefits [2].

Although the information on tumors is vast and valuable, there are certain concerns regarding the management of HCC, particularly in terms of the limited ability to perform repeat biopsies and sampling errors. Localized antitumor immune responses cannot exist without continuous communication with the periphery. Therefore, a thorough understanding of the immune responses to cancer must include the role of the peripheral immune system, in addition to that within the TME [9]. In addition, as peripheral blood sampling is readily available, minimally invasive, and repeatable, the use of blood-based immune biomarkers can compensate for the above-mentioned limitations of tissue-based immune biomarkers during cancer immunotherapy [10]. Recent studies have shown that peripheral T cells with memory phenotypes exert relatively superior antitumor effects. In advanced melanoma treated with anti-CTLA4, a higher proportion of CD45RO+ memory cells relative to total CD8+ T cells in blood was a positive predictor of both response rate and overall survival (OS) [11]. In advanced melanoma and non-small cell lung cancer patients, a high CD45RA− to CCR7+ central memory T (TCM) cell to Teff (TCM/Teff) ratio in blood was shown to be a positive predictor of both response to anti-PD1 therapy and survival [12]. Considering that inflamed tumors have high TCM/Teff ratios in the blood compared to non-inflamed tumors suggests the importance of TCM cells in anti-tumor immune responses in patients treated with ICIs [12]. Another study including metastatic melanoma patients demonstrated that patients who responded to therapy had larger clones (those occupying >0.5% of the repertoire) of effector memory T (TEM) cells than non-responding patients or controls after treatment. Further, the 6-month clinical response to an ICI (pembrolizumab or ipilimumab plus nivolumab combination therapy) was associated with a large CD8+ T cell clone count 21 days after treatment, suggesting that post-ICI peripheral CD8+ clonality can provide information regarding the likely long-term treatment response in metastatic melanoma patients [13].

However, the association between peripheral immune cell profiles and response to Atez/Bev treatment in HCC patients is not yet well known. In this study, we focused on the blood T cell subset that is considered to play a major role in cancer immunity and performed flow cytometry analysis of peripheral blood mononuclear cells (PBMCs) at baseline and after 3 weeks of Atez/Bev treatment to evaluate the status and dynamics of peripheral T cell subpopulations in HCC patients receiving Atez/Bev treatment and to explore biomarkers that are predictive of therapeutic response.

## 2. Materials and Methods

### 2.1. Patients

Eighty-three patients who received Atez/Bev treatment for HCC at Hiroshima University Hospital between October 2020 and June 2022 and for whom cryopreserved pre-treatment PBMCs were available were retrospectively enrolled in this study. After subjecting the PBMCs to fluorescence-activated cell sorting (FACS), 70 patients were found to have sufficient cell counts with >10,000 CD3+ T cells. Cryopreserved PBMCs from 3 weeks after treatment that also met the above criteria were available in 51 of the 70 patients. PBMC cryopreservation and FACS were also performed using blood samples collected from 18 healthy volunteers as a control group.

To reduce selection bias, the study included all cases where treatment intervention was possible within a specific time period, and for whom obtaining consent and sample collection were feasible.

### 2.2. Treatment Regimens

Patients received 1200 mg of atezolizumab plus 15 mg/kg body weight of bevacizumab intravenously every 3 weeks. Drug treatment was continued until the occurrence of unacceptable toxic effects or loss of clinical benefit. Patients who transiently or permanently discontinued either atezolizumab or bevacizumab because of an adverse event were allowed to continue single-agent therapy, as long as the attending physician determined that there was clinical benefit. Patients who received other treatments, such as transcatheter arterial chemoembolization (TACE) during Atez/Bev treatment were censored.

### 2.3. Clinical and Laboratory Assessments

**Figure 1:**
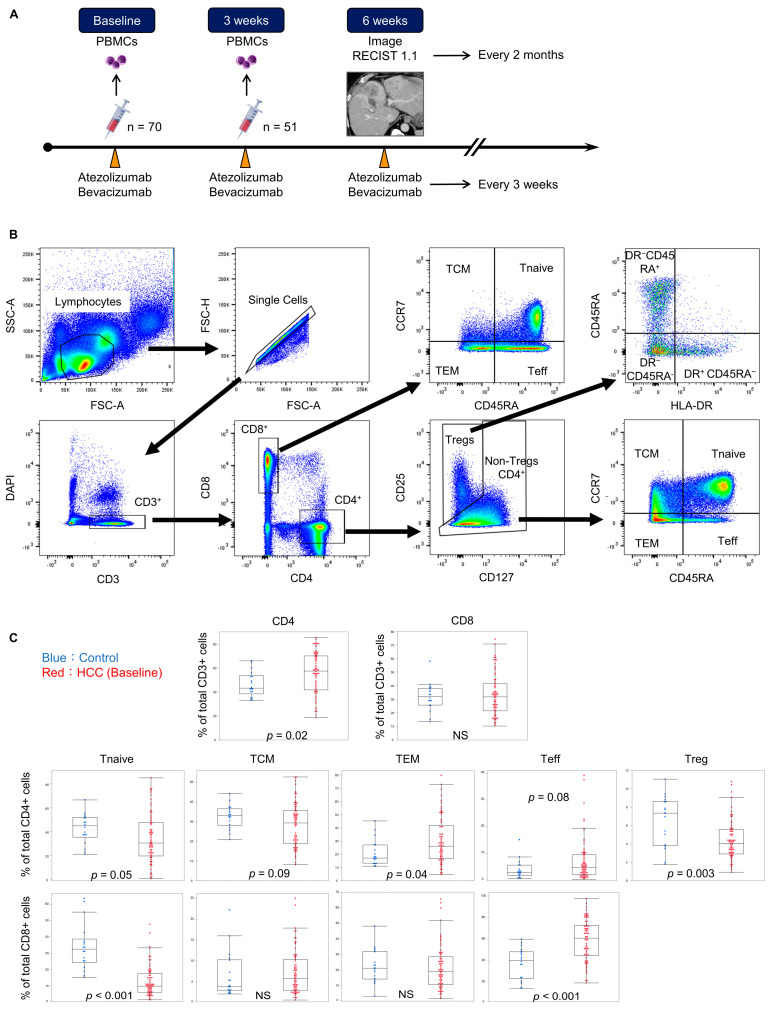
(**A**) Study design and timing of comparison between HCC and healthy control groups. Timeline of PBMC collection and objective response evaluation. Paired samples at baseline and 3 weeks post-treatment were analyzed in 51 patients. Schemes were created with BioRender.com, https://www.biorender.com/ (accessed on 24 February 2023). (**B**) Gating strategy to define T cell subpopulations in PBMCs. (**C**) Box plots showing the proportion of T cell subpopulations at baseline. The blue and red dots represent the values in individuals in the healthy control and HCC groups, respectively.

Clinical and laboratory assessments were performed before Atez/Bev treatment. Objective response was evaluated using response evaluation criteria in solid tumors (RECIST) [14] after 6 weeks (2.7–16.0) of treatment and every 2 months thereafter. The timeline of PBMC collection and objective response evaluation is shown in Figure 1A. Progression-free survival (PFS) was defined as the period from the initiation of treatment until disease progression, specifically identified as progressive disease (PD) according to RECIST evaluation, death, or the last patient contact, whichever occurred first. Overall survival (OS) was defined as the duration from the initiation of treatment until death or the last patient contact.

### 2.4. PBMCs

PBMCs were collected from each patient at baseline and at 3 weeks (1.7–11.4) after the initiation of Atez/Bev treatment. Briefly, 20 mL of peripheral venous blood was collected using a heparinized syringe. PBMCs were isolated by Ficoll gradient centrifugation using a SepMate-50 tube (STEMCELL, Vancouver, BC, Canada) according to the manufacturer’s instructions. For cryopreservation, PBMC pellets were resuspended in an appropriate volume of chilled resuspension medium (40% FBS in RPMI) to achieve a concentration of 20 × 10^6^ cells/mL, and an equivalent volume of chilled 2× freezing medium (30% DMSO in RPMI containing 40% FBS) was added to achieve a concentration of 10 × 10^6^ cells/mL according to the 10× Genomics Single Cell protocol. The PBMCs resuspended with freezing medium were placed in a freezing container (BICELL) and were placed overnight in a −80 °C freezer, and then transferred to liquid nitrogen for storage for later use.

### 2.5. Flow Cytometry

The PBMC cryovials were removed from storage and immediately thawed in a water bath at 37 °C. For analysis of the cell surface molecules, PBMC suspensions were prepared and incubated with the following antibodies: PreCP-Cyanine 5.5-anti-human CD3 (317336, Biolegend, San Diego, CA, USA), FITC-anti-human CD4 (357406, Biolegend, San Diego, CA, USA), APC-Cyanine 7-anti-human CD8 (344714, Biolegend, San Diego, CA, USA), PE-anti-human CD25 (302606, Biolegend, San Diego, CA, USA), PE-Cyanine 7-anti-human CD45RA (304126, Biolegend, San Diego, CA, USA), APC-anti-human CD127 (351316, Biolegend, San Diego, CA, USA), BV421TM-anti-human CCR7 (353208, Biolegend, San Diego, CA, USA) and BV785-anti-human HLA-DR (307624, Biolegend, San Diego, CA, USA). Flow cytometry data were acquired using a BD LSR Fortessa X-20 cell analyzer (BD Biosciences, San Jose, CA, USA) and analyzed with FlowJo software version 10.10.0 (Tree Star, OR, USA). The cells were sorted into CCR7+CD45RA+ (Tnaïve), CCR7−CD45RA+ (Teff), CCR7+CD45RA− (TCM), CCR7−CD45RA− (TEM), and CD4+CD25+CD127− (Treg) subpopulations, as shown in the gating strategy (Figure 1B). Since it is difficult to make comparisons based on absolute counts, the analysis of subpopulations was based on the percentage of each population. Spanning Tree Progression of Density Normalized Events (SPADE) analysis [15] was implemented using the R package.

### 2.6. Immunohistochemistry (IHC)

To assess the relationship between the immune profile of peripheral T cell subsets and tumor tissue immune classes, CD8 IHC staining was performed using baseline tumor biopsy tissues in 10 patients. After deparaffinization of the paraffin-embedded HCC biopsy tissues and rehydration with graded ethanol washes, heat-induced antigen retrieval was performed for 15 min, followed by incubation with the CD8 monoclonal antibody (Anti-CD8 alpha) overnight at 4 °C after pretreatment of tissues with 0.3% hydrogen peroxide. The sections were then washed and incubated with rabbit IgG (SignalStain Boost IHC Detection Reagent #8114) for 30 min at room temperature, after which the sections were washed and incubated for 5 min at room temperature with DAB working solution (SignalStain DAB Substrate Kit #8059, Cell Signaling Technology, Danvers, MA, USA). The sections were counterstained with hematoxylin and eosin and mounted after dehydration and attainment of transparency. Tumor areas in biopsy tissue were mapped by a pathologist, and the number of tumor-infiltrating lymphocytes (TILs) expressing CD8 were counted and quantified at 400× magnification using the color deconvolution function of ImageJ version 1.53t [16]. Assessment of intra-tumor lymphocyte infiltration was independently performed by one pathologist and two hepatologists who were blinded to the patients’ clinical courses.

### 2.7. Statistical Analysis

Intergroup differences in continuous and categorical variables between two groups were tested using the Mann–Whitney U test and Fisher’s exact test, respectively. Each quantitative variable was divided into two groups based on the median. Comparison of PFS after Atez/Bev treatment was assessed using Kaplan–Meier curves (for the entire follow-up period), the log-rank test, and multivariate Cox regression hazard models. The Wilcoxon signed-rank test was used to compare paired differences for continuous values. Statistical analysis was performed using JMP Pro14.0.0 (SAS Institute Inc., Cary, NC, USA). Kaplan–Meier curves were illustrated using the open-source R programming software, EZR on R26, version 1.55. *p* < 0.05 was considered statistically significant.

## 3. Results

### 3.1. Clinical Characteristics

Supplementary Table S1 summarizes the clinical characteristics of the study cohort. The cohort consisted of 55 males and 15 females with a median age of 73.5 years, who were observed for a period of 10.6 (1.7–26.7) months. Among them, 44 patients exceeded the up-to-seven criteria [17]. The control group consisted of 18 healthy persons without liver disease (14 males and 4 females) with a median age of 36.5 years. Median PFS and OS of the HCC patients were 5.25 months (0.7–25.4) and 10.7 months (1.7–13.6), respectively. Thirty-four patients died during the observation period, and one patient died from ruptured esophageal varices, which was the only cause of death other than HCC.

### 3.2. Lower CD8+ Tnaïve and Higher CD8+ Teff Proportion at Baseline in HCC Patients Compared to Controls

Figure 1C shows a comparison of CD4+ and CD8+ T cell subpopulations in the HCC patients at baseline versus those in controls. The proportion of CD8+ Tnaïve cells at baseline was significantly lower in HCC patients compared to controls (*p* < 0.001). On the other hand, the proportion of CD8+ Teff cells was significantly higher at baseline in HCC patients compared to the control group (*p* < 0.001). In addition, the Treg proportion was significantly lower in patients with HCC compared to controls (*p* = 0.003).

### 3.3. Relationship between Patient Background Characteristics and T Cell Subpopulation

The correlation between patient background characteristics and baseline T cell subpopulation was examined using the Mann–Whitney U test and Kruskal–Wallis test (Supplementary Table S2). CD8+ TCM proportion was higher in patients with larger tumor size (*p* = 0.03), those with a greater number of tumors (*p* = 0.02), and those who exceeded the up-to-seven criteria (*p* = 0.02). Further, contrary to CD8+ TCM, the CD8+ Teff proportion was lower in patients with a high tumor burden (tumor size: *p* = 0.07, number of tumors: *p* = 0.005, and those who exceeded the up-to-seven criteria: *p* = 0.02).

### 3.4. Higher CD8+ TCM Proportion before Treatment Prolongs PFS

**Figure 2:**
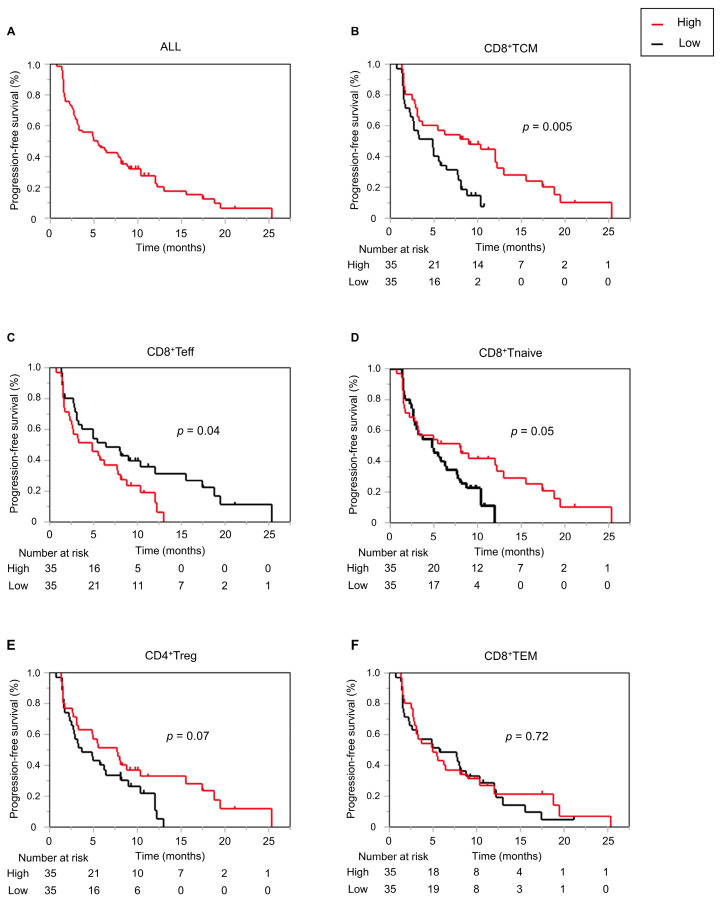
Kaplan–Meier curves representing progression-free survival in all patients (**A**) and according to baseline CD8+ TCM (**B**), CD8+ Teff (**C**), CD8+ Tnaïve (**D**), CD4+ Treg (**E**), and CD8+ TEM (**F**) cell proportions.

The percentages of CD8+ T cell subpopulation to total CD8+ T cell count and CD4+ T cell subpopulation to total CD4+ T cell count at baseline were calculated, and patients were then stratified into high and low groups based on their respective median values. Notably, the group exhibiting high CD8+ TCM displayed a significantly longer PFS (4.8 months [95%CI: 2.27–5.76] vs. 9.0 months [95%CI: 3.09–12.24] in low versus high groups, respectively, *p* = 0.005). Furthermore, contrary to the CD8+ TCM cell proportion, PFS was shorter in the group with a high CD8+ Teff proportion (6.4 months [95%CI: 3.09–12.07] vs 4.8 months [95%CI: 2.27–7.70] in low versus high groups, respectively, *p* = 0.04). Although prolonged PFS was observed in the high CD8+ Tnaïve group (4.8 months [95%CI: 2.73–6.44] vs. 8.0 months [95%CI: 2.76–12.99], *p* = 0.05) and high CD4+ Treg group (7.7 months [95%CI: 3.09–10.39] vs. 3.7 months [95%CI: 2.5–6.45], *p* = 0.07), the level did not reach statistical significance. There was no difference in PFS in terms of CD8+ TEM proportion between the high and low groups at baseline (5.6 months [95%CI: 2.27–8.75] vs. 5.0 months [95%CI: 2.89–8.13], *p* = 0.72) (Figure 2). Representative flow cytometry figures of high and low groups for CD8+ Tnaive, TCM, and Teff proportions are presented in Supplementary Figure S1. Multivariate analysis with CD8+ TCM, CD8+ Teff, and Child-Pugh score as covariates, which have been previously reported as factors related to PFS [18], revealed that high CD8+ TCM correlated significantly with PFS (Table 1).

### 3.5. Increase in CD8+ TEM Proportions and Decrease in CD8+ Teff Proportions in the First 3 Weeks in the Response Group

**Figure 3:**
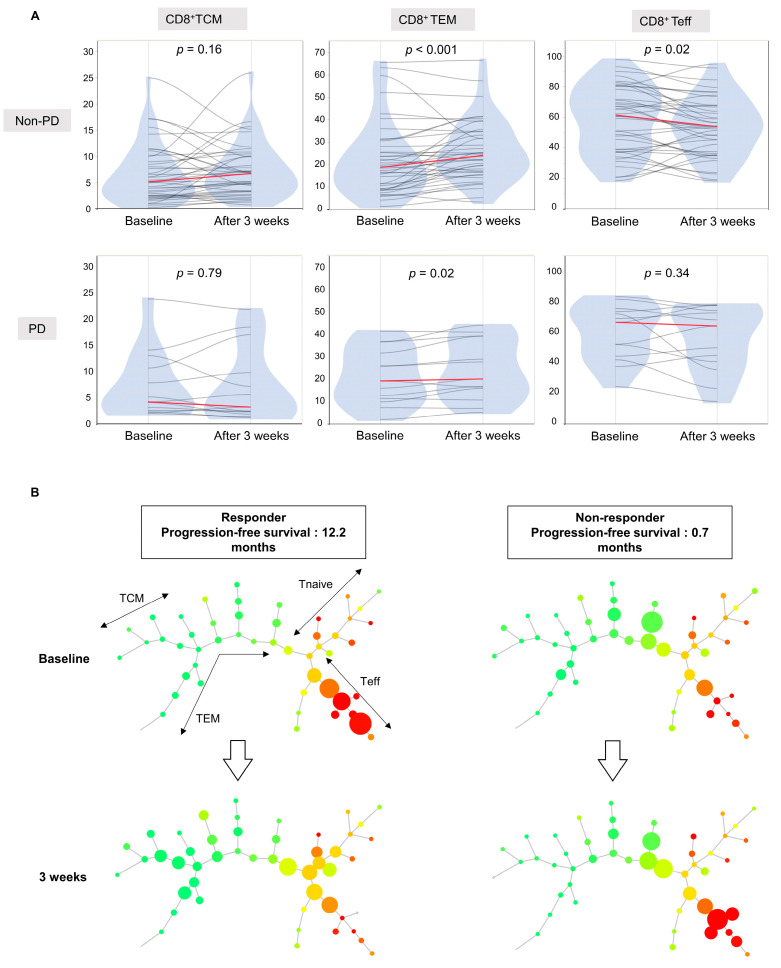
(**A**) Violin plots representing the changes in peripheral T cell subpopulations of TCM, TEM, and Teff at baseline and 3 weeks after treatment. Each line connects the values before and after treatment in the same patient. *p*-values were determined by the Wilcoxon signed-rank test. (**B**) SPADE-generated maturation profiles using CD8 cells extracted by gating in FlowJo software version 10.10.0. Representative figures for treatment responders and non-responders are shown. All samples were down-sampled to 1000 cells to ensure a uniform circle size. CD45RA expression is shown in red/green color scale.

**Figure 4:**
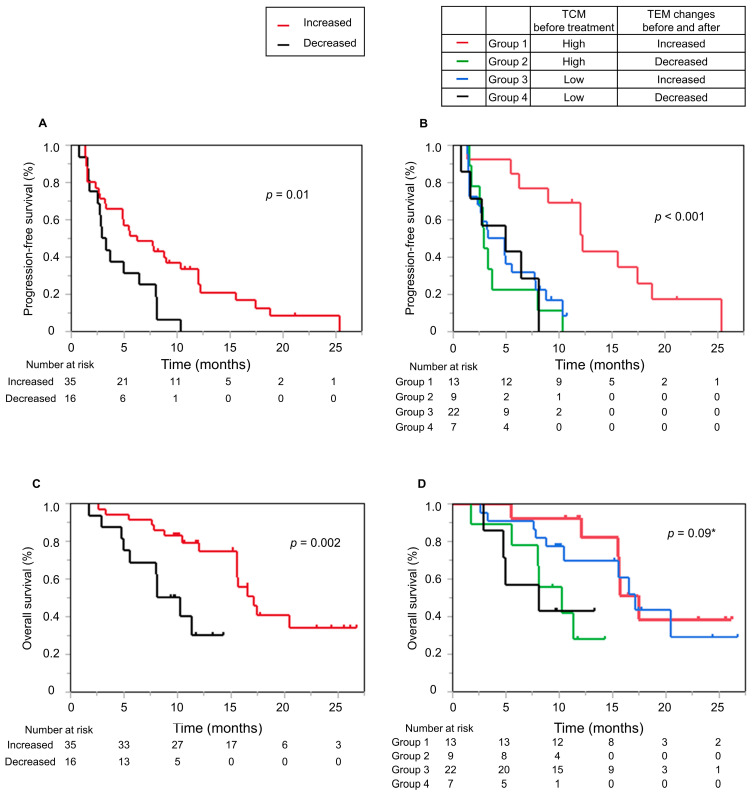
(**A**) Kaplan–Meier curves presenting progression-free survival (PFS) according to changes in CD8+ TEM cells after treatment. (**B**) Kaplan–Meier curves presenting PFS according to the combination of baseline TCM and changes in CD8+ TEM proportion after treatment. (**C**) Kaplan–Meier curves presenting overall survival (OS) according to changes in CD8+ TEM after treatment, and (**D**) the combination of baseline TCM and changes in CD8+ TEM after treatment. * *p*-values were calculated by comparing groups 3 and 4.

Subsequently, we examined changes in the proportion of CD8+ T cell subpopulations in groups subdivided according to disease progression into PD and non-PD groups based on the initial treatment response. As shown in Figure 3A, the CD8+ TEM proportion increased (*p* < 0.001) and the CD8+ Teff proportion decreased significantly after 3 weeks in the non-PD group (*p* = 0.02). Representative flow cytometry figures for the CD8+ EMT and Teff increase and decrease groups before and after treatment are presented in Supplementary Figure S2. We applied SPADE to the flow cytometry data to visually assess CD8+ T cell dynamics with treatment. Arranging the SPADE results in the order of increasing PFS confirmed that clusters of CD8+ TCM and CD8+ Teff subpopulations showed opposite changes in most patients with prolonged PFS of over 10 months (Supplementary Figure S3). Figure 3B shows representative SPADE images of responders with an increase in CD8+ TCM and a decrease in CD8+ Teff clusters and non-responders with no increase in CD8+ TCM and no decrease in CD8+ Teff clusters. Furthermore, we found that PFS was significantly prolonged in the group with an increased CD8+ TEM proportion at 3 weeks (3.1 months [95%CI: 1.68–6.45] vs. 6.3 months [95%CI: 3.26–10.39], *p* = 0.01) (Figure 4A). With respect to clinical characteristics, patients with an increased CD8+ TEM proportion after treatment had a fewer number of tumors (*p* = 0.009), were within up-to-seven criteria (*p* = 0.01), and had lower des-γ-carboxy prothrombin (DCP) (*p* = 0.04) levels at baseline (Supplementary Table S3).

### 3.6. Patients with High CD8+ TCM Cell Proportions at Baseline and Increased CD8+ TEM Cell Proportions after Treatment Have Prolonged PFS

Based on the above findings, we hypothesized that the combination of baseline CD8+ TCM cell proportion and change in CD8+ TEM cell proportion with treatment might be a high-precision biomarker for predicting PFS. To evaluate this, the patients were divided into four groups according to baseline CD8+ TCM cell proportion and the change in CD8+ TEM cell proportion with treatment (Figure 4B). The evaluation showed that patients with a high CD8+ TCM proportion at baseline who experienced an increase in CD8+ TEM proportion with treatment had significantly longer PFS (*p* < 0.001) (Figure 4B). Conversely, the group with high CD8+ TCM proportion at baseline but no increase in CD8+ TEM proportion with treatment showed no difference in PFS compared with the group with low CD8+ TCM at baseline.

### 3.7. Patients with Increased CD8+ TEM Cell Proportions after Treatment Have Prolonged OS

We also examined the impact of peripheral T cell subpopulations on OS. Although we could not find a clear relationship between peripheral T cell subpopulations and OS alone, OS was significantly prolonged in the group with increased CD8+ TEM proportion 3 weeks after treatment compared to the group with no increase in CD8+ TEM proportion (9.2 months [95%CI: 5–NA] vs. 17.1 months [95%CI: 15.53–NA], *p* = 0.002) (Figure 4C). In addition, we examined the four groups as with the analysis of PFS and found that, unlike PFS, OS was also prolonged in the group with low CD8+ TCM at baseline and increased CD8+ TEM proportion after treatment (17.1 months [95%CI: 10.49–NA] vs. 8.1 months [95%CI: 2.86–NA], *p* = 0.09) (Figure 4D). Child-Pugh grade B, exceeding up-to-seven criteria, and high baseline DCP levels were associated with poor OS in univariate analysis (Supplementary Figure S4). Among these factors, multivariate analysis revealed that increased CD8+ TEM proportion (*p* = 0.006, HR (95%CI): 0.26 (0.10–0.67)) and Child-Pugh B (*p* = 0.03, HR (95%CI): 2.56 (1.09–6.02)) were significant risk factors for poor OS (Table 2).

### 3.8. Correlation between Intratumoral CD8+ T Cell Infiltration and the Proportions of Peripheral CD8+ T Cell Subpopulations

**Figure 5:**
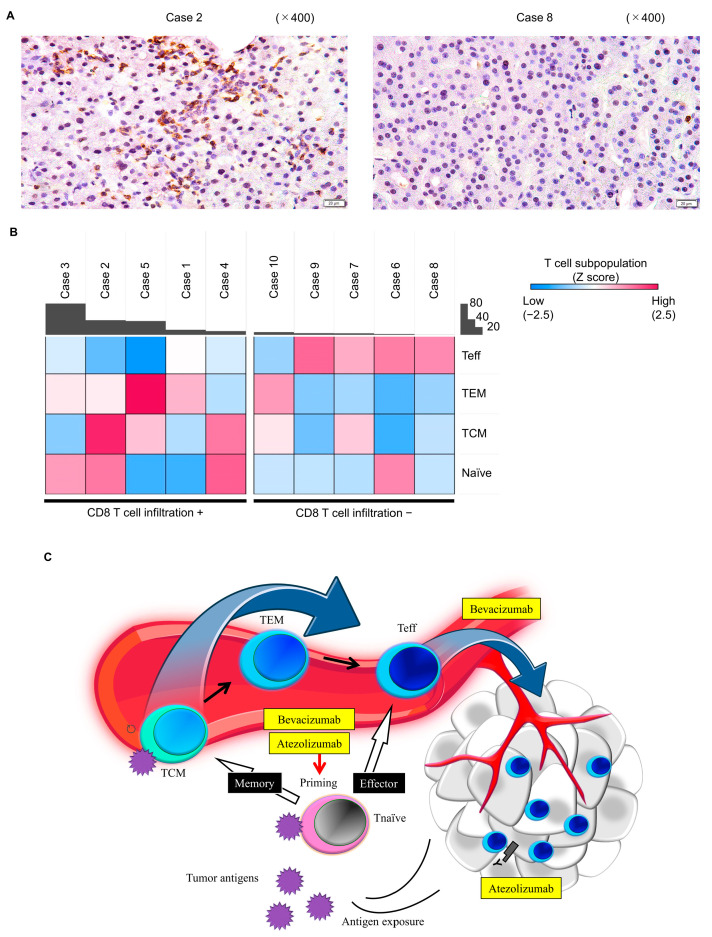
(**A**) Representative high-power field views of the results of immunohistochemistry staining of CD8+ cells in the tumors in two patients. (**B**) Heat map summarizing the correlation between peripheral T cell subpopulations and pathological intratumoral CD8+ T cell infiltration. Red and blue colors represent the subpopulations of peripheral T cells. A Z-score normalized with respect to T cell subset was used to generate the heatmap, to visualize differences between patients. The black bar indicates the mean number of positive cells per field of view (356 × 190 μm) obtained at 400× magnification from three fields. (**C**) A possible hypothetical model explaining the correlation between peripheral T cell subpopulation dynamics and responsiveness to immunotherapy. Schemes were created using BioRender.com, https://www.biorender.com/ (accessed on 24 February 2023).

After IHC for baseline CD8 expression in HCC tissues, the resultant numbers were quantified using ImageJ software version 1.53t (Figure 5A). Ten patients were then divided into two groups based on the median value; those with values above the median were placed in the CD8 T cell infiltration positive (+) group and those with values below the median were placed in the CD8 T cell infiltration negative (−) group. A heat map summarizing the correlation between peripheral CD8+ T cell subpopulations and pathological intratumoral CD8+ T cell infiltration (Figure 5B) suggested that intratumoral CD8+ T cell negative patients tended to have a high peripheral CD8+ Teff proportion, and intratumoral CD8+ T cell positive patients tended to have low peripheral CD8+ Teff and high memory T cell proportions.

## 4. Discussion

Here, we reported that higher CD8+ TCM proportion at baseline is associated with tumor inflammation in HCC, as well as with longer PFS in response to Atez/Bev treatment for HCC, especially in patients with an increase in CD8+ TEM cell proportion 3 weeks after treatment. As far as we know, this is the first report evaluating the phenomenon of dynamics in circulating T cell subpopulations with Atez/Bev treatment for HCC. Cancer is a systemic disease, and prolonged inflammation is a hallmark of cancer. Hence, the localized antitumor immune response cannot exist without continuous communication with the periphery.

PBMCs from HCC patients in this study presented a lower CD8+ Tnaïve proportion and a higher CD8+ Teff proportion compared to control samples. These findings are consistent with the profile in patients with non-small cell lung carcinoma, advanced head and neck squamous cell carcinoma, and autoimmune diseases [12,19,20]. Based on these previous results, we considered our cryopreserved PBMC samples as being suitable for the current analysis because the differences in PBMC profiles were attributed to persistent antigen stimulation, including that associated with tumors.

The association between a high CD8+ TCM proportion at baseline and improved treatment outcomes is consistent with reports that TCM cells are the primary repository of the immunogenic experiences of a lifetime [21,22]. TCM cells are characterized by their lymph node-homing properties and exhibit a higher proliferative capacity than their TEM counterparts [23]. There is previous evidence supporting the hypothesis that an inverse relationship between the circulating proportion of CD8+ Teff cells and the tumor-infiltrating CD8+ T cells, as shown by IHC, is indicative of the presence of terminally differentiated T cells that are unable to reach the tumor [12].

We considered the following three possibilities to explain the observed dynamics in the peripheral T cell subpopulation after treatment: (1) differentiation of CD8+ TCM cells re-exposed to cancer antigens; (2) rescue of exhausted CD8+ T cells and increase in the infiltration of intratumoral CD8+ T cells; and (3) promotion of the T cell priming phase. A possible hypothetical model for the relationship between the dynamics of the peripheral T cell subpopulation and responsiveness to immunotherapy is shown in Figure 5C. We were able to demonstrate that the higher the CD8+ TCM proportion, the higher the number of intratumoral CD8+ T cells tended to be. Zhu et al. reported that patients with a high density of intratumoral CD8+ T cells showed longer OS (HR = 0.29, *p* = 0.001) and PFS (HR = 0.54, *p* = 0.053) with Atez/Bev compared to sorafenib [2]. Hence, a greater anti-tumor effect of Atez/Bev can be expected in the group with a high CD8+ TCM proportion. It is possible that CD8+ TCM are re-exposed to cancer antigens released due to the anti-tumor effect of Atez-Bev, which induces their differentiation into CD8+ TEM cells. Antibodies targeting PD-L1 rescue T cells from their exhausted status, and anti-VEGF augments intratumoral T cell infiltration, potentially through vascular normalization and endothelial cell activation [24]. A previous report showed that intra-tumoral CD8+ T cells increased following Atez/Bev treatment [25]. Considering the reports of the inverse relationship between the frequency of Teff cells in circulation and the inflammation signature in non-small cell lung cancer [12] and the relationship between intratumoral CD8+ T cells and peripheral CD8+ Teff in the current study, the decrease in peripheral CD8+ Teff after treatment might be a phenomenon of this positive anti-tumor change. A previous in vivo study showed that the promotion of priming of T cells by CTLA4 blockade predominantly induces a proliferative signature in a subset of transitional memory T cells [26,27,28]. Peripheral memory T population dynamics in Atez/Bev treatment might also reflect the results of promoted priming. Surprisingly, patients with an increased CD8+ TEM proportion tended to have prolonged OS, regardless of their baseline CD8+ TCM proportion. Some studies have shown that tumor-specific TEM responses can remain functional against cancer even in settings of persistent antigen exposure [29]. In tremelimumab plus durvalumab treatment, the promotion of priming by CTLA4 blockade is an important pharmacological effect that significantly improved OS versus that with sorafenib, although PFS was not significantly different in advanced HCC patients [30]. These results suggest that OS might be affected by the function of memory T cells in immunotherapy.

In our study, there was a tendency for baseline CD8+ TCM proportion and the dynamics of CD8+ TEM proportion after treatment to be related to tumor burden. A higher tumor burden was associated with a higher CD8+ TCM proportion at baseline and also with a lesser increase in CD8+ TEM proportion after treatment. For the prediction of prognosis, the effects of high CD8+ TCM proportion as a favorable marker and high tumor burden as an unfavorable marker negate each other. Therefore, treatment-induced dynamics might be more important in predicting the effect of immunotherapy than baseline immune profiles. The observed result that the group with a higher CD8+ TCM proportion at baseline but no increase in CD8+ TEM proportion after treatment had a similar PFS as the group with a lower CD8+ TCM proportion at baseline also supports this consideration.

Some investigations have proposed that NASH-HCC is less responsive to immunotherapy [31,32]. However, in our study, there were no significant differences between NASH and non-NASH in terms of both OS (20.5 months [95%CI: 12.5-NA] vs. 15.6 months [95%CI: 10.3–17.4], *p* = 0.21) and PFS (6.0 months [95%CI: 3.1–10.4] vs. 4.9 months [95%CI: 2.6–8.8], *p* = 0.49). Other previous reports have shown consistent results with our study [33,34]. Notably, a previous report stated that ICIs are more likely to be effective in NASH-HCC when combined with a molecular-targeted agent, such as bevacizumab [35].

Various papers have reported the importance of T cell diversity [36,37]. We reanalyzed our previous repertoire analysis data to examine the relationship between T cell diversity and gene expression levels of immune-related genes in tumors (the datasets generated during that study are available in the Figshare repository: https://doi.org/10.6084/m9.figshare.22215760.v3: accessed on 31 January 2024) [38]. We found no clear correlation between T cell receptor (TCR) repertoire profiling and the expression of immune cell markers in tumors (Supplementary Figure S5). In our opinion, these results suggest that TCR repertoire profiling can provide complementary information to the immune cell population and gene expression profiling.

In a study by Fairfax et al. that examined the characteristics of peripheral CD8+ T cells in relation to ICI in patients with metastatic melanoma, they explored the properties of the peripheral TCR repertoire for association with clinical outcomes [13]. They reported no association between clonal diversity and clinical outcomes, although the number of large clones (defined as clones with count numbers >0.5% of the total number of clones per chain) was particularly high in the response group. They further stated that patients with a higher number of large clones after treatment had a predominantly longer PFS and OS and that there was a strong correlation between the number of large clones and the number of CD8+ TEM. The observed results of our study, that an increase in the percentage of TEM cells before and after treatment has a positive impact on both PFS and OS, is supported by the report of Fairfax et al. We showed that even the simple assessment of percentage change in CD8+ TEM cells can be used to assess treatment response and might be a useful predictor of response. A strength of the observed novel relationship between the increase in CD8+ TEM large 3 weeks after treatment and outcome is that it is agnostic to the target, similar to the observation made by Fairfax et al. These results suggest that analyzing not only the population of T cells and their infiltration into tumors but also performing TCR repertoire profiling, might lead to the discovery of new markers.

However, since analyzing T cell distribution is not easy in real-world clinical practice at present, we considered if there might be any serum protein marker that can serve as a surrogate for the T cell subpopulation. We previously analyzed the serum levels of 17 cytokines/chemokines using multiplex Luminex assay in 204 advanced HCC patients who had never received systemic therapy and were registered in the GEO database. The data are accessible through the GEO Series accession number: GSE261672 (https://www.ncbi.nlm.nih.gov/geo/query/acc.cgi?acc=GSE261672). Fifty-seven of those cases who received Atez/Bev treatment right after the cytokine measurements were included in the present study cohort. The evaluation demonstrated no apparent correlation between baseline cytokine status and baseline TCM, which we considered to be indicative of responsiveness to Atez/Bev treatment (Supplementary Figure S6). Although there were correlations between some cytokines and T cell subsets, they were not exact, suggesting the need for further study.

Studies evaluating the results of therapy demonstrate how the peripheral immune response is regulated and dysregulated during effective or ineffective immune responses. However, the mechanisms driving many of these features remain unknown. Thus, future studies will also need to provide mechanistic insights into peripheral immune reorganization in order to enable the design of therapeutic strategies that restore the disrupted immune system to a healthy homeostatic immune setpoint. Understanding these mechanisms will contribute to immunotherapeutic strategies for the primary treatment of HCC.

Several limitations associated with the present study warrant mention. First, although the study included all patients who had received Atez/Bev treatment during the target period, the number of patients was small because it was a single-center study and the treatment was new. Second, the appropriate sample size required for dividing the total cohort into test and validation cohorts could not be met, resulting in insufficient validation. Third, we were not able to examine additional surface markers due to sample volume limitations. For example, CD8+/PD-1+ HCC populations reportedly tend to be associated with prolonged PFS and OS with Atez/Bev treatment compared to other populations [2]. Therefore, consideration of these surface markers might be warranted in the future. Even so, since this study was not designed to identify a completely new biomarker but rather to validate markers that have been reported in other cancers for HCC, the study itself served as a validation test of existing candidate biomarkers.

## 5. Conclusions

Baseline CD8+ TCM proportion is a good indicator of the efficacy of Atez/Bev therapy for HCC and might be a promising early predictor of prolonged OS with therapy.

## Figures and Tables

**Table 1:** Multivariate analysis of factors potentially related to PFS after initiation of Atez/Bev treatment.

	Univariate Analysis	Multivariate Analysis	
	*p* Value	HR (95% CI)	*p* Value
Age (<74 vs. ≥74 years)	0.33		
Sex (Male vs. Female)	0.32		
Etiology (HBV vs HCV vs. NBNC)	0.07		
CD4+ Tnaïve * (<30.28 vs. ≥30.28)	0.49		
CD4+ TCM * (<29.37 vs. ≥29.37)	0.3		
CD4+ TEM * (<26.15 vs. ≥26.15)	0.15		
CD4+ Teff* (<4.50 vs. ≥4.50)	0.74		
Treg * (<4.09 vs. ≥4.09)	0.07		
CD8+ Tnaïve * (<9.95 vs. ≥9.95)	0.05		
CD8+ TCM * (<5.72 vs. ≥5.72)	0.005	0.44 (0.24–0.79)	0.006
CD8+ TEM * (<19.34 vs. ≥19.34)	0.71		
CD8+ Teff * (<60.59 vs. ≥60.59)	0.04	1.28 (0.67–2.44)	0.46
Child–Pugh score ** (B vs A)	0.53	1.65 (0.83–3.26)	0.15
mALBI grade (1 vs. 2a/vs. b)	0.28		
Serum AFP value (<36.2 vs. ≥36.2), ng/mL	0.54		
Serum DCP value (≥328.5 vs. <328.5), mAU/mL	0.99		
Up-to-seven criteria (out vs. in)	0.8		
History of systemic treatment (with vs. without)	0.36		
History of TACE treatment (yes vs. no)	0.76		
Metastasis (yes vs. no)	0.29		

* CD4+ and CD8+ subpopulation data are percentages of the total CD4+ and CD8+ cell population. ** No significant differences.

**Table 2:** Multivariate analysis of factors potentially related to OS after initiation of Atez/Bev treatment.

	Univariate Analysis	Multivariate Analysis	
	*p* Value	HR (95% CI)	*p* Value
Age (<74 vs. ≥74 years)	0.19		
Sex (Male vs. Female)	0.49		
Etiology (HBV vs. HCV vs. NBNC)	0.15		
CD4+ Tnaïve * (<30.28 vs. ≥30.28)	0.88		
CD4+ TCM * (<29.37 vs. ≥29.37)	0.19		
CD4+ TEM * (<26.15 vs. ≥26.15)	0.7		
CD4+ Teff * (<4.50 vs. ≥4.50)	0.85		
Treg * (<4.09 vs. ≥4.09)	0.31		
CD8+ Tnaïve * (<9.95 vs. ≥9.95)	0.97		
CD8+ TCM * (<5.72 vs. ≥5.72)	0.74		
CD8+ TEM * (<19.34 vs. ≥19.34)	0.1		
CD8+ Teff * (<60.59 vs. ≥60.59)	0.28		
Post-Pre CD8+ TEM (Increased vs. decreased)	0.002	0.26 (0.10–0.67)	0.006
Child–Pugh score (B vs. A)	0.01	2.56 (1.09–6.02)	0.03
mALBI grade (1 vs. 2a vs. 2b)	0.14		
Serum AFP value (<36.2 vs. ≥36.2), ng/mL	0.12		
Serum DCP value (≥328.5 vs. <328.5), mAU/mL	0.02	1.15 (0.47–2.82)	0.75
Up-to-seven criteria (out vs. in)	0.01	2.4 (0.89–6.50)	0.08
History of systemic treatment (with vs. without)	0.58		
History of TACE treatment (yes vs. no)	0.79		
Metastasis (yes vs. no)	0.86		

* CD4+ and CD8+ subpopulation data are percentages of the total CD4+ and CD8+ cell population.

## Data Availability

The data and material used and/or analyzed in the current study are available from the corresponding author, A.O., on reasonable request, and will not include confidential patient information. The datasets used in this study are available in the Figshare repository: https://doi.org/10.6084/m9.figshare.22215760.v3: accessed on 31 January 2024 [38] and GEO Series accession number GSE261672 (https://www.ncbi.nlm.nih.gov/geo/query/acc.cgi?acc=GSE261672).

## Supplementary Materials

The following supporting information can be downloaded at: https://www.mdpi.com/article/10.3390/cancers16071328/s1, Figure S1: Representative flow cytometry figures of high and low CD8 positive-Tnaive, -TCM, and -Teff cell groups; Figure S2: Representative flow cytometry figures for increase and decrease in CD8+ EMT and Teff proportion groups before and after treatment; Figure S3: Maturity profiles of all patients generated by SPADE using CD8 cells extracted by gating in FlowJo software version 10.10.0. All samples were down-sampled to 1000 cells to ensure a uniform circle size; Figure S4: Kaplan-Meier curves of overall survival according to Child-Pugh class, up-to-seven criteria and des-γ-carboxy prothrombin (DCP) levels; Figure S5: Correlation between gene expression levels of immune cell markers and diversity of repertoire. The black bars represent the diversity indexes (Pielou’s evenness, Shannon-Weaver index H’ and Inv. Simpson’s index 1/λ) obtained from repertoire analysis. Shannon, Shannon-Weaver index H’; Simpson, Inv. Simpson’s index 1/λ; Pielou, Pielou’s evenness; Figure S6: Scatter plots showing the correlation between serum cytokine levels and the proportions of T cell subsets; Table S1: Descriptive characteristics of patients who received Atez/Bev therapy for HCC at Hiroshima University Hospital; Table S2: Correlation between patient background factors and T cell subpopulations; Table S3: Correlations between the clinical characteristics of the patients and increase or decrease in CD8+TEM proportion.
