# Supplementary material for: Peripheral T Cell Subpopulations as a Potential Surrogate Biomarker during Atezolizumab plus Bevacizumab Treatment for Hepatocellular Carcinoma
Source: Cancers (Basel). 2024 Mar 28;16(7):1328. doi: 10.3390/cancers16071328 (PMC11011052; doi:10.3390/cancers16071328)
Supplement: Supplementary file 1 [file cancers-16-01328-s001.zip › Supplementary Figure S2.pptx]

## Slide 1
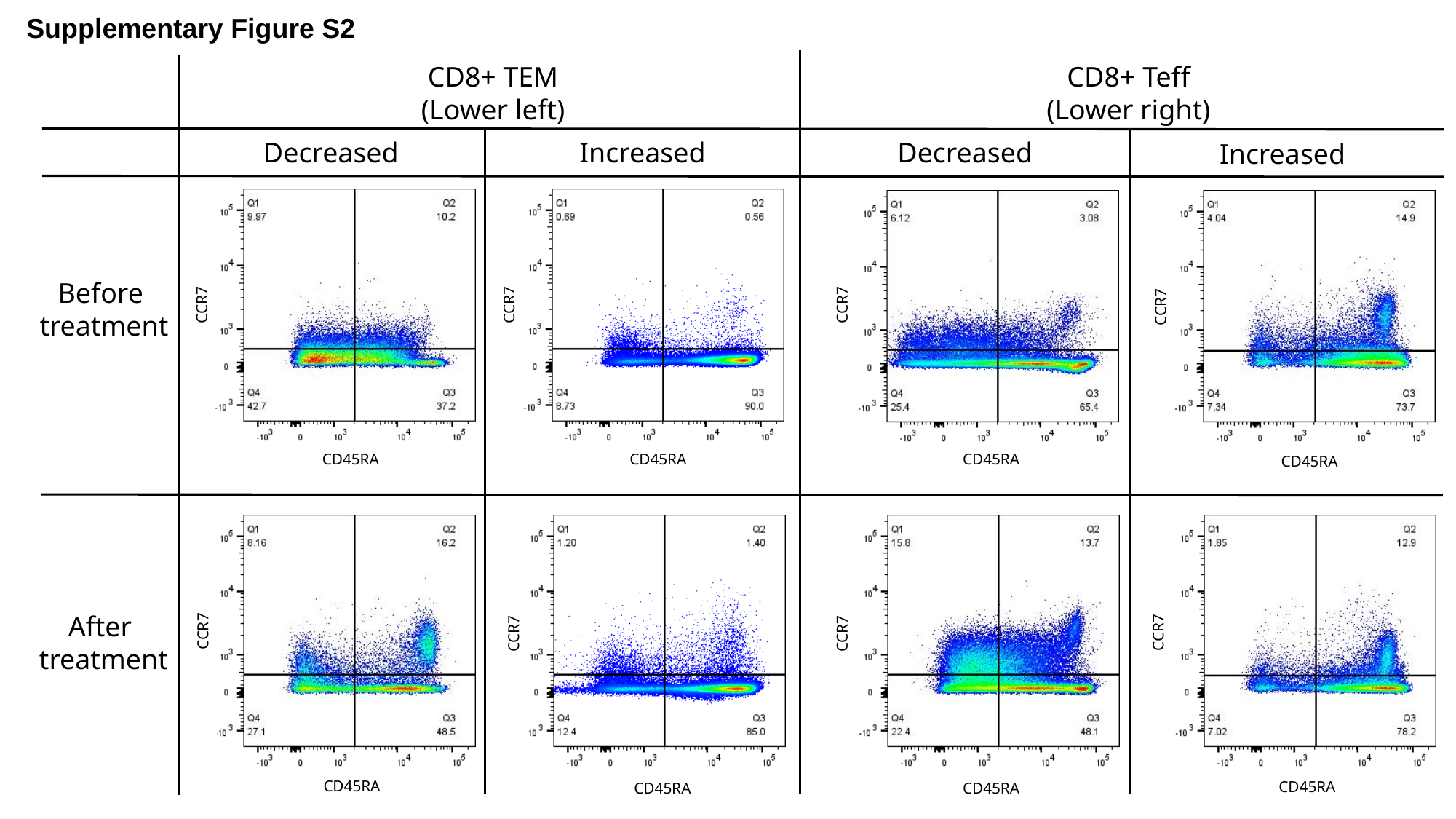

Supplementary Figure S2
CD8+ TEM
(Lower left)
CD8+ Teff
(Lower right)
Decreased
Increased
Decreased
Increased
Before
treatment
CCR7
CCR7
CCR7
CCR7
CD45RA
CD45RA
CD45RA
CD45RA
After
treatment
CCR7
CCR7
CCR7
CCR7
CD45RA
CD45RA
CD45RA
CD45RA
