# Supplementary material for: Peripheral T Cell Subpopulations as a Potential Surrogate Biomarker during Atezolizumab plus Bevacizumab Treatment for Hepatocellular Carcinoma
Source: Cancers (Basel). 2024 Mar 28;16(7):1328. doi: 10.3390/cancers16071328 (PMC11011052; doi:10.3390/cancers16071328)
Supplement: Supplementary file 1 [file cancers-16-01328-s001.zip › Supplementary Figure S4.pptx]

## Slide 1
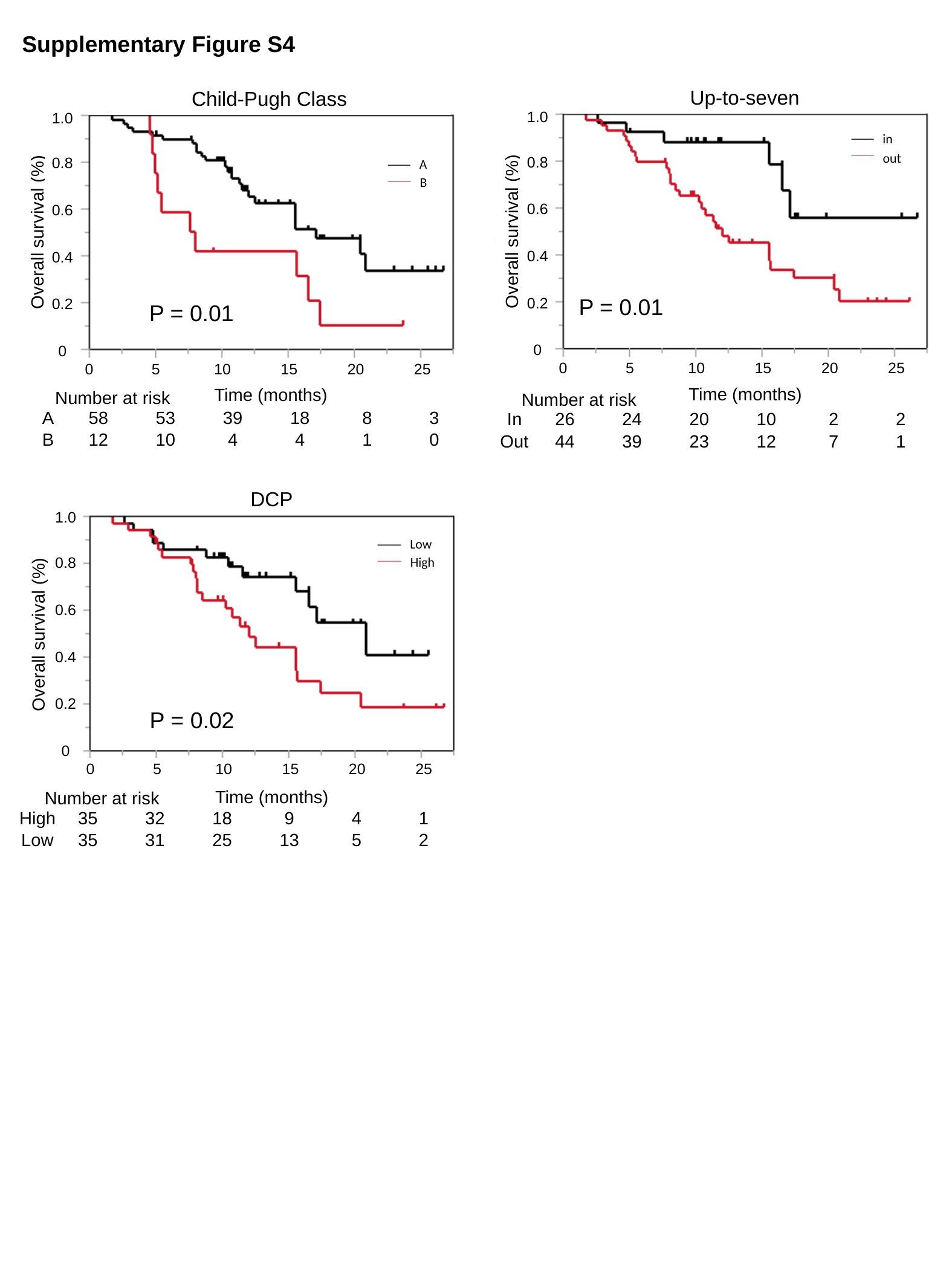

Supplementary Figure S4
Up-to-seven
Child-Pugh Class
| 1.0 |
| --- |
| 0.8 |
| 0.6 |
| 0.4 |
| 0.2 |
| 0 |
| 1.0 |
| --- |
| 0.8 |
| 0.6 |
| 0.4 |
| 0.2 |
| 0 |
in
out
A
B
Overall survival (%)
Overall survival (%)
P = 0.01
P = 0.01
| 0 | | 5 | | 10 | | 15 | | 20 | | 25 |
| --- | --- | --- | --- | --- | --- | --- | --- | --- | --- | --- |
| 0 | | 5 | | 10 | | 15 | | 20 | | 25 |
| --- | --- | --- | --- | --- | --- | --- | --- | --- | --- | --- |
Time (months)
Time (months)
| Number at risk | Number at risk | | | | | | | | | | |
| --- | --- | --- | --- | --- | --- | --- | --- | --- | --- | --- | --- |
| A | 58 | | 53 | | 39 | | 18 | | 8 | | 3 |
| B | 12 | | 10 | | 4 | | 4 | | 1 | | 0 |
| Number at risk | Number at risk | | | | | | | | | | |
| --- | --- | --- | --- | --- | --- | --- | --- | --- | --- | --- | --- |
| In | 26 | | 24 | | 20 | | 10 | | 2 | | 2 |
| Out | 44 | | 39 | | 23 | | 12 | | 7 | | 1 |
DCP
| 1.0 |
| --- |
| 0.8 |
| 0.6 |
| 0.4 |
| 0.2 |
| 0 |
Low
High
Overall survival (%)
P = 0.02
| 0 | | 5 | | 10 | | 15 | | 20 | | 25 |
| --- | --- | --- | --- | --- | --- | --- | --- | --- | --- | --- |
Time (months)
| Number at risk | Number at risk | | | | | | | | | | |
| --- | --- | --- | --- | --- | --- | --- | --- | --- | --- | --- | --- |
| High | 35 | | 32 | | 18 | | 9 | | 4 | | 1 |
| Low | 35 | | 31 | | 25 | | 13 | | 5 | | 2 |
