# Supplementary material for: Peripheral T Cell Subpopulations as a Potential Surrogate Biomarker during Atezolizumab plus Bevacizumab Treatment for Hepatocellular Carcinoma
Source: Cancers (Basel). 2024 Mar 28;16(7):1328. doi: 10.3390/cancers16071328 (PMC11011052; doi:10.3390/cancers16071328)
Supplement: Supplementary file 1 [file cancers-16-01328-s001.zip › Supplementary Figure S6.pptx]

## Slide 1
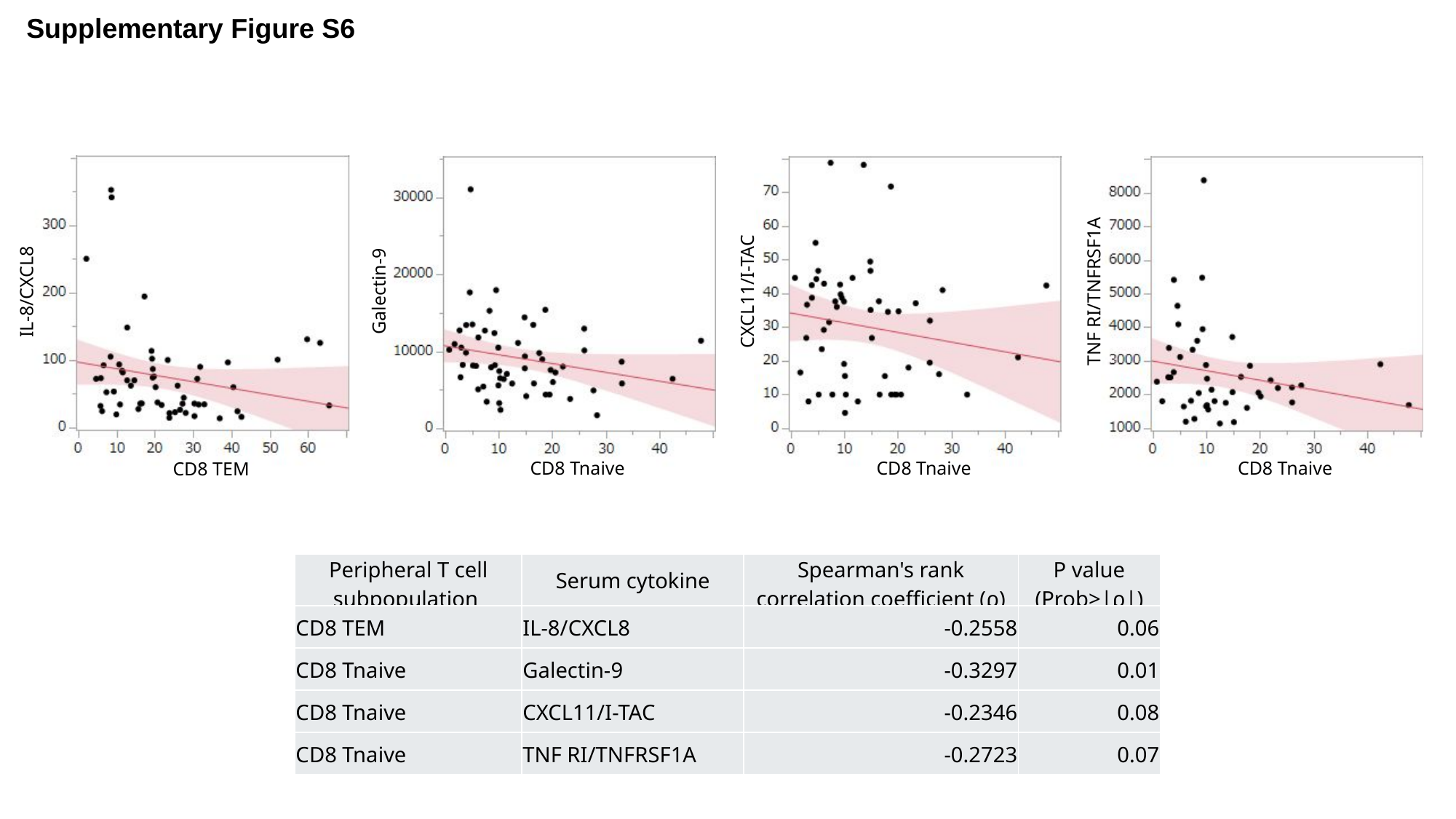

Supplementary Figure S6
IL-8/CXCL8
Galectin-9
TNF RI/TNFRSF1A
CXCL11/I-TAC
CD8 Tnaive
CD8 Tnaive
CD8 Tnaive
CD8 TEM
| Peripheral T cell subpopulation | Serum cytokine | Spearman's rank correlation coefficient (ρ) | P value (Prob>|ρ|) |
| --- | --- | --- | --- |
| CD8 TEM | IL-8/CXCL8 | -0.2558 | 0.06 |
| CD8 Tnaive | Galectin-9 | -0.3297 | 0.01 |
| CD8 Tnaive | CXCL11/I-TAC | -0.2346 | 0.08 |
| CD8 Tnaive | TNF RI/TNFRSF1A | -0.2723 | 0.07 |
