# Supplementary figures and images for: Peripheral T Cell Subpopulations as a Potential Surrogate Biomarker during Atezolizumab plus Bevacizumab Treatment for Hepatocellular Carcinoma
Source: Cancers (Basel). 2024 Mar 28;16(7):1328. doi: 10.3390/cancers16071328 (PMC11011052; doi:10.3390/cancers16071328)

## Slide 1
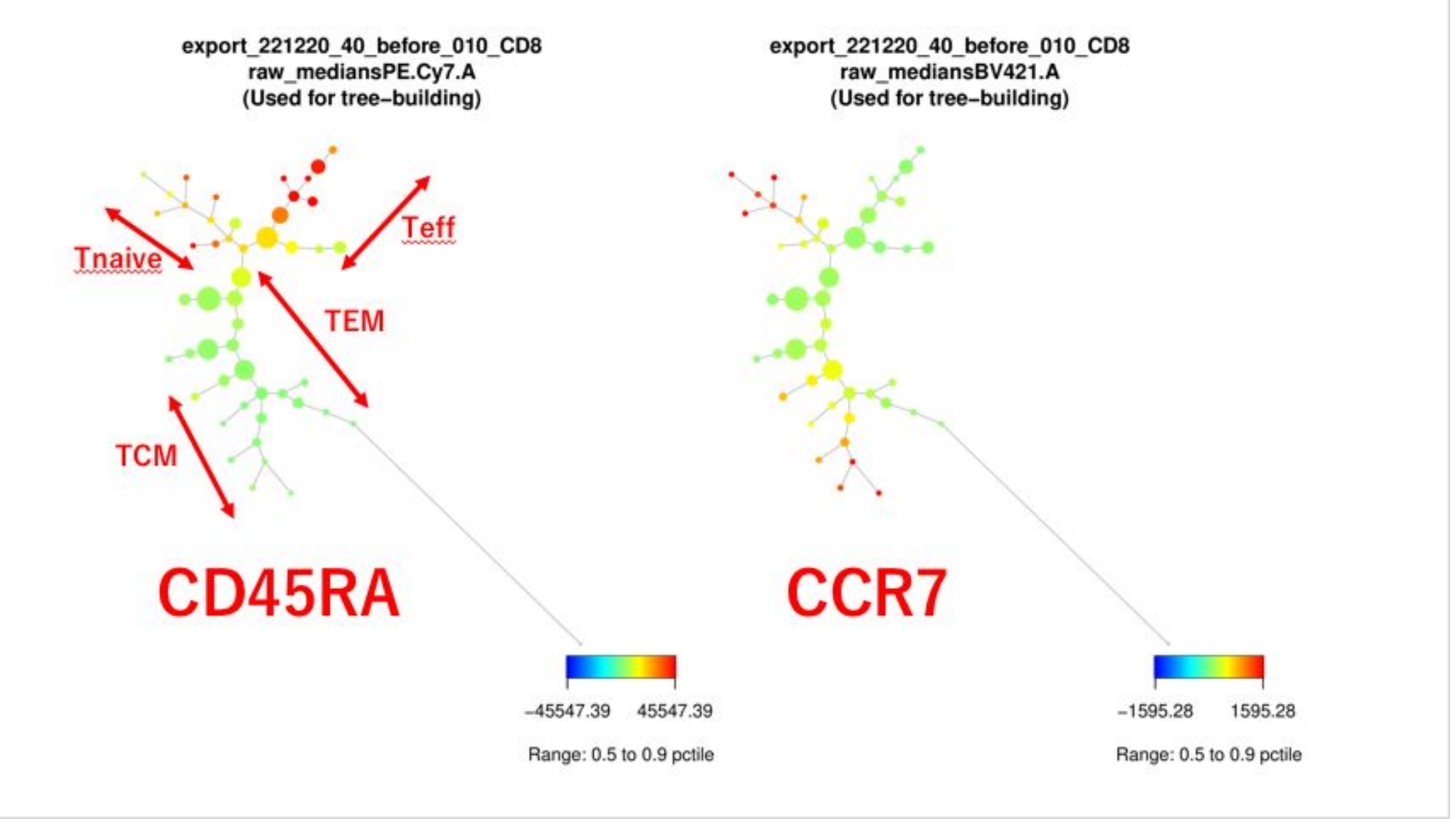

#

## Slide 2
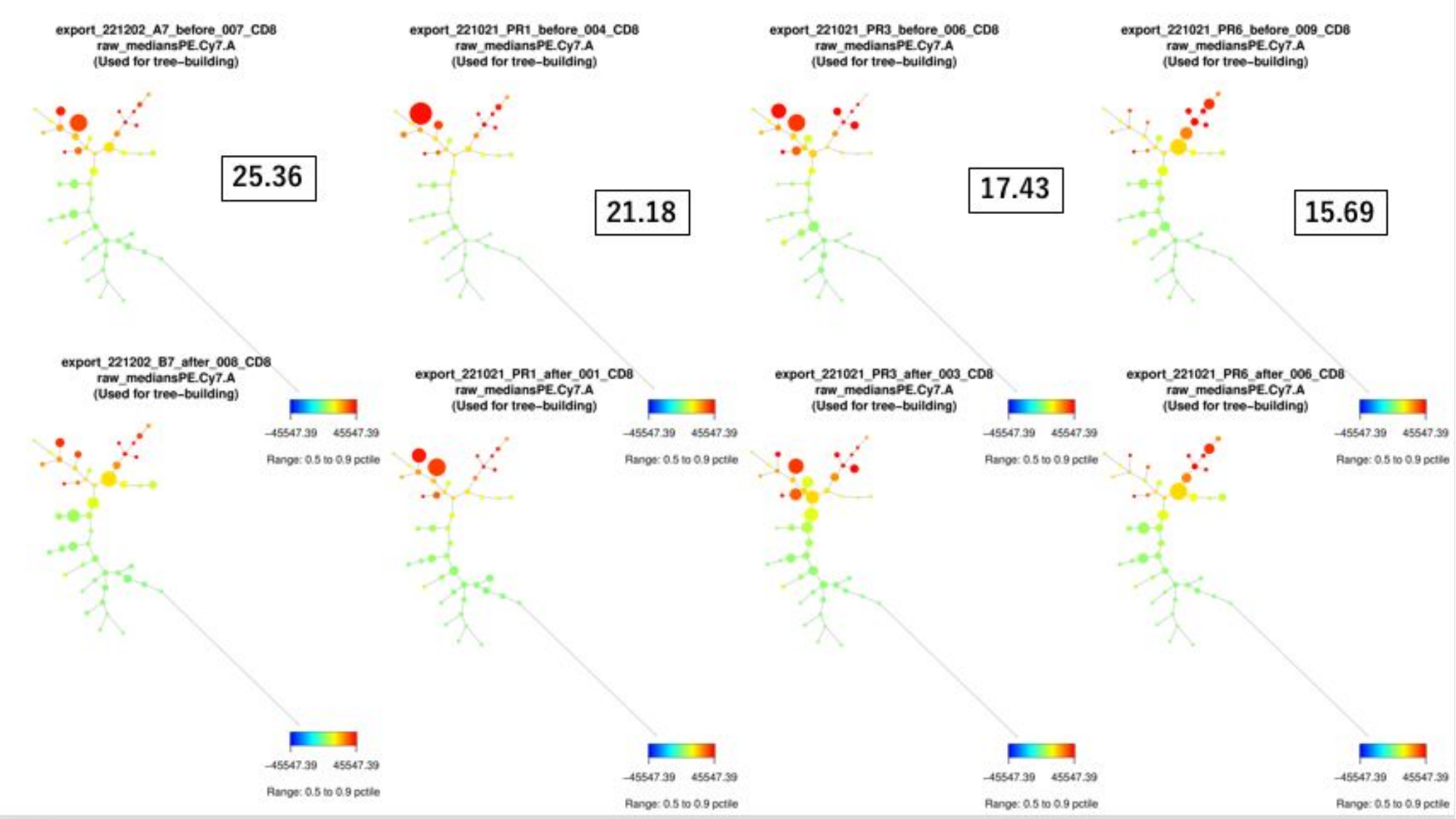

#

## Slide 3
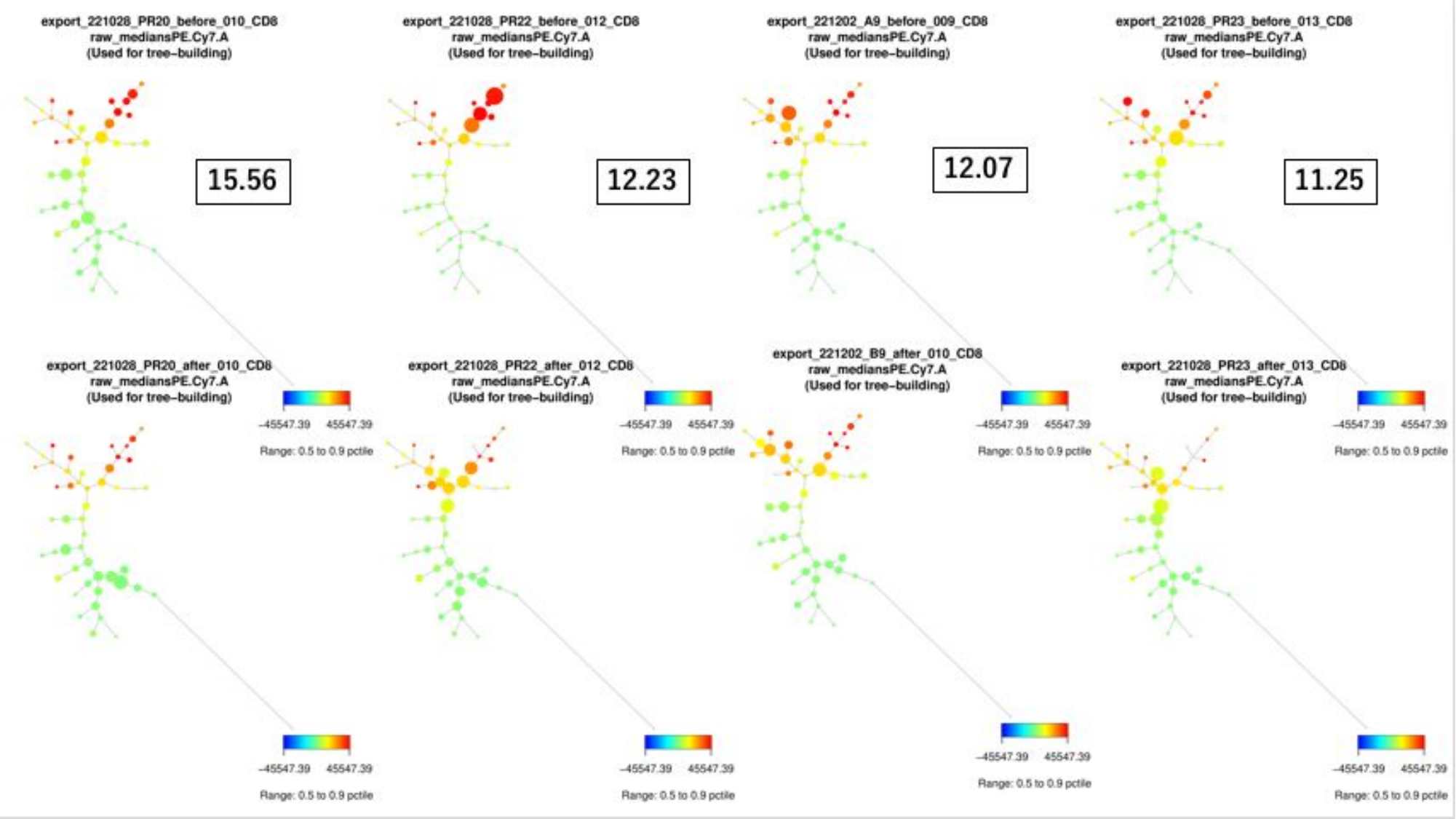

#

## Slide 4
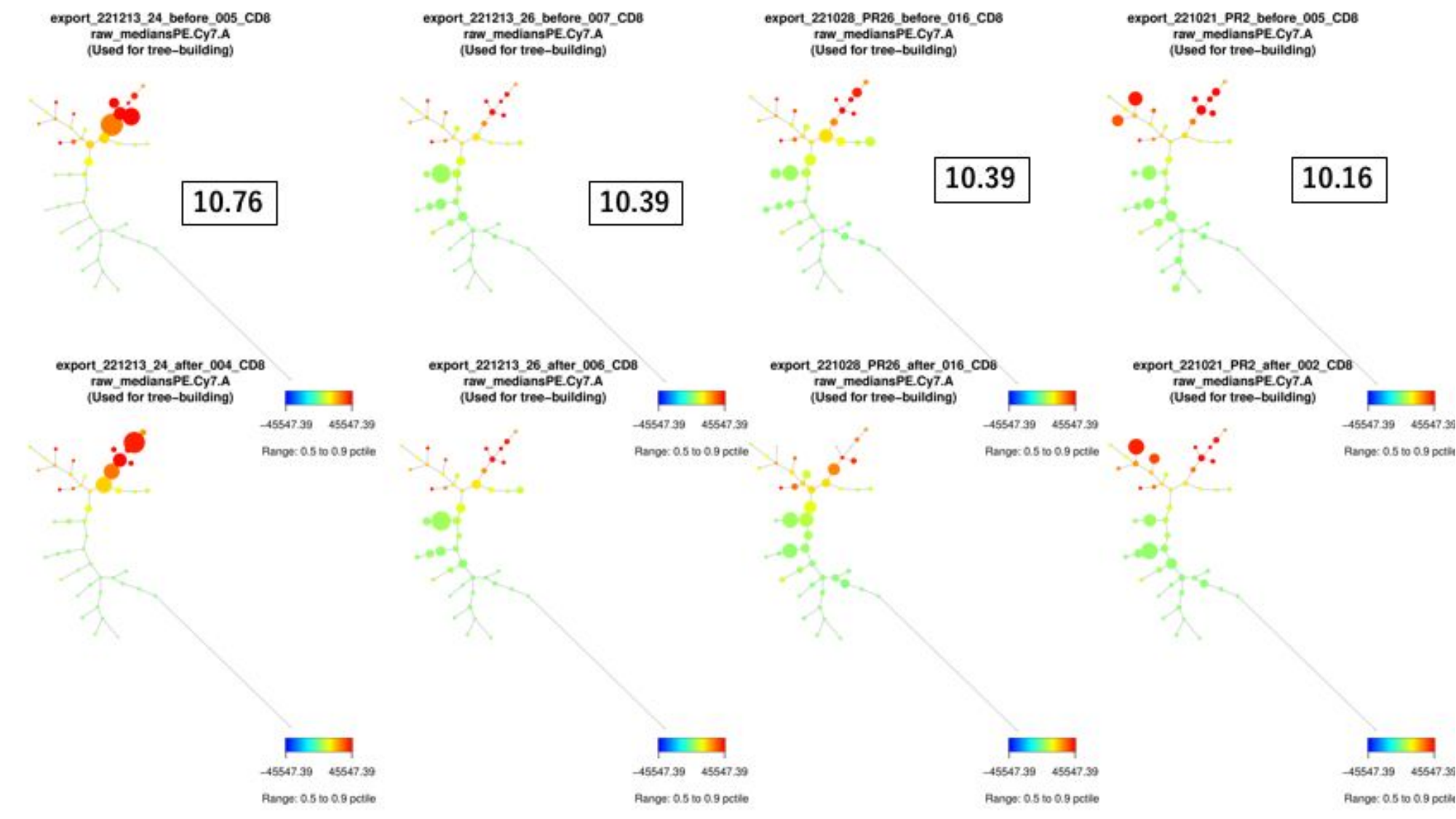

#

## Slide 5
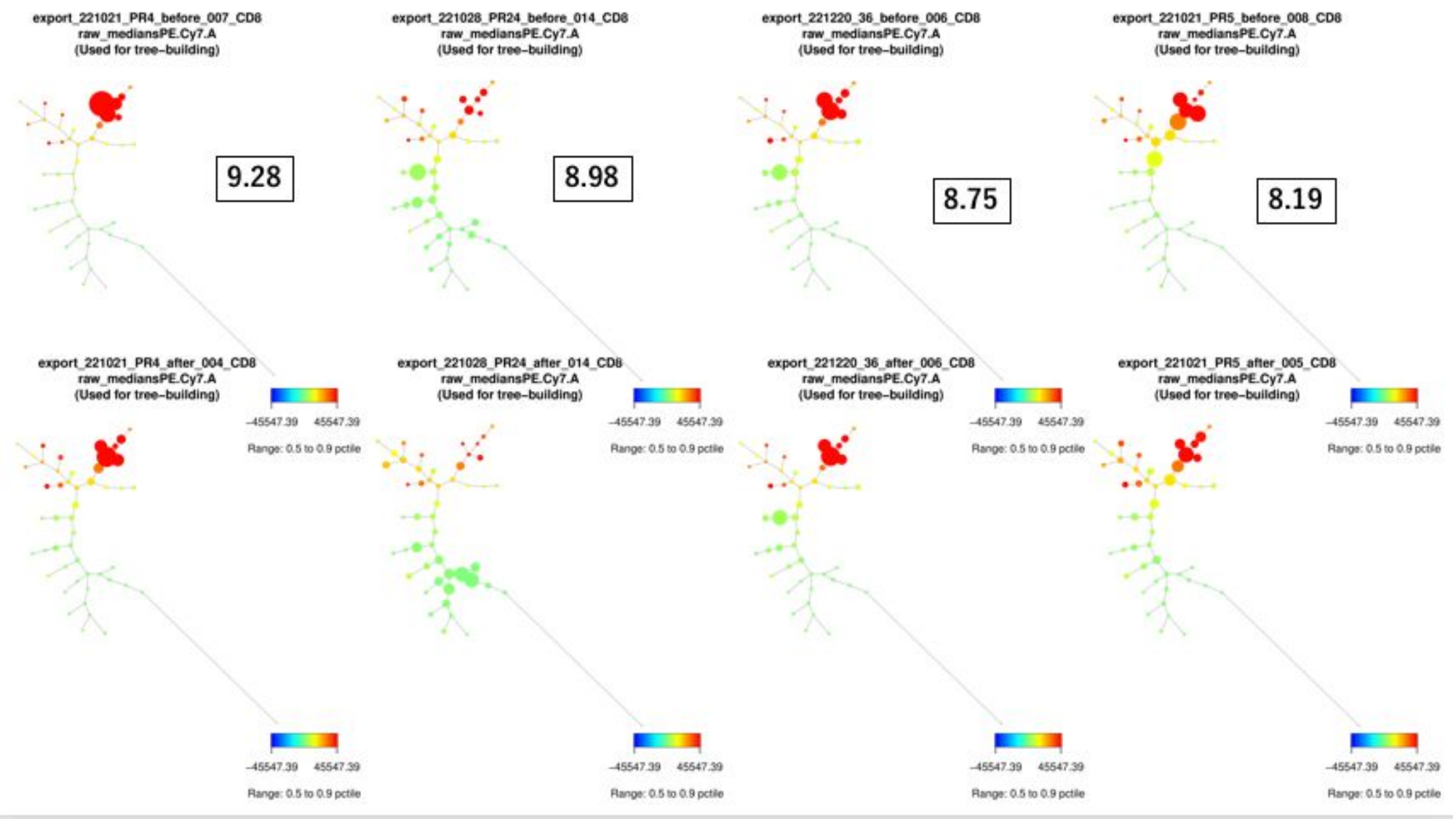

#

## Slide 6
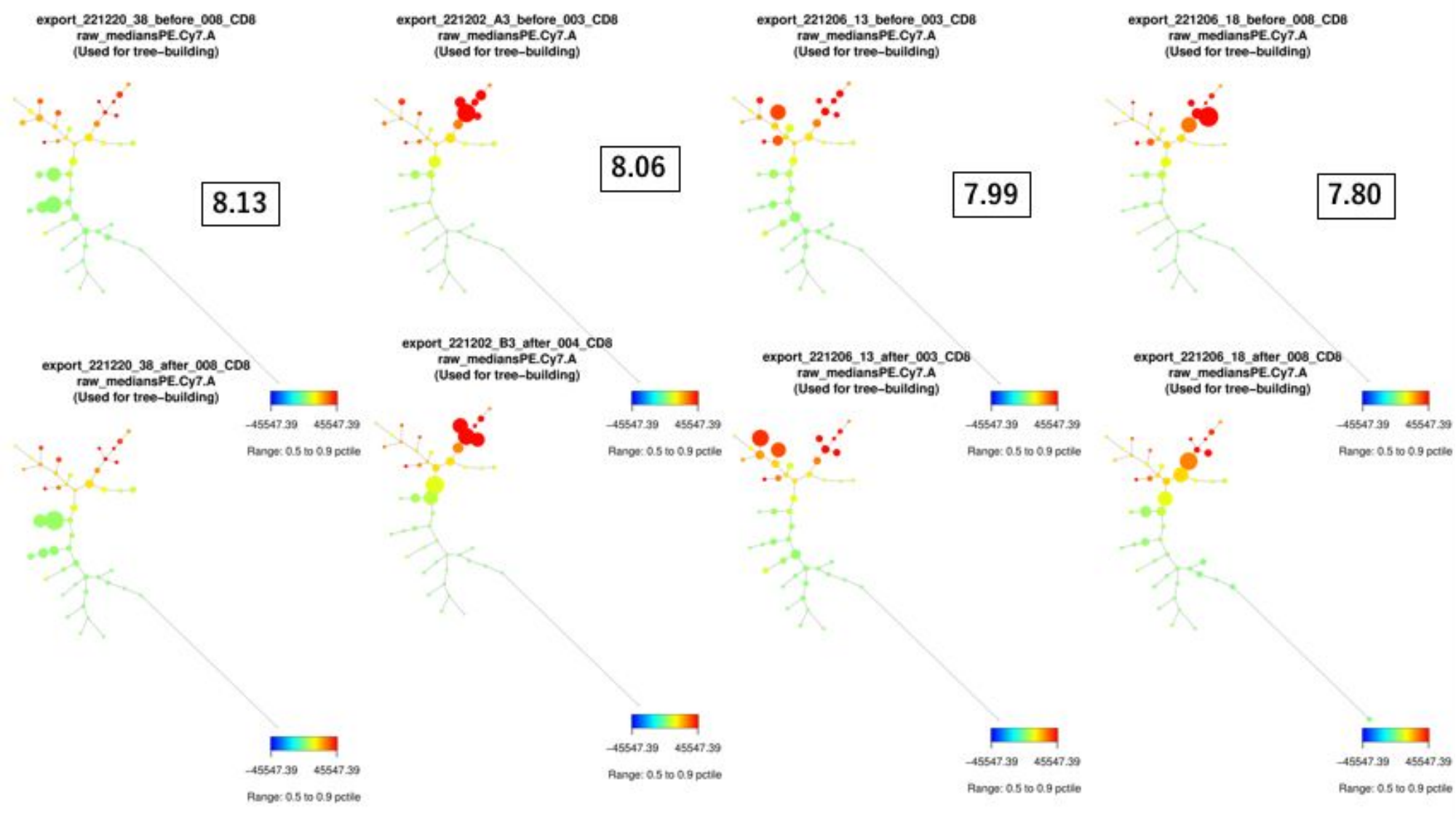

#

## Slide 7
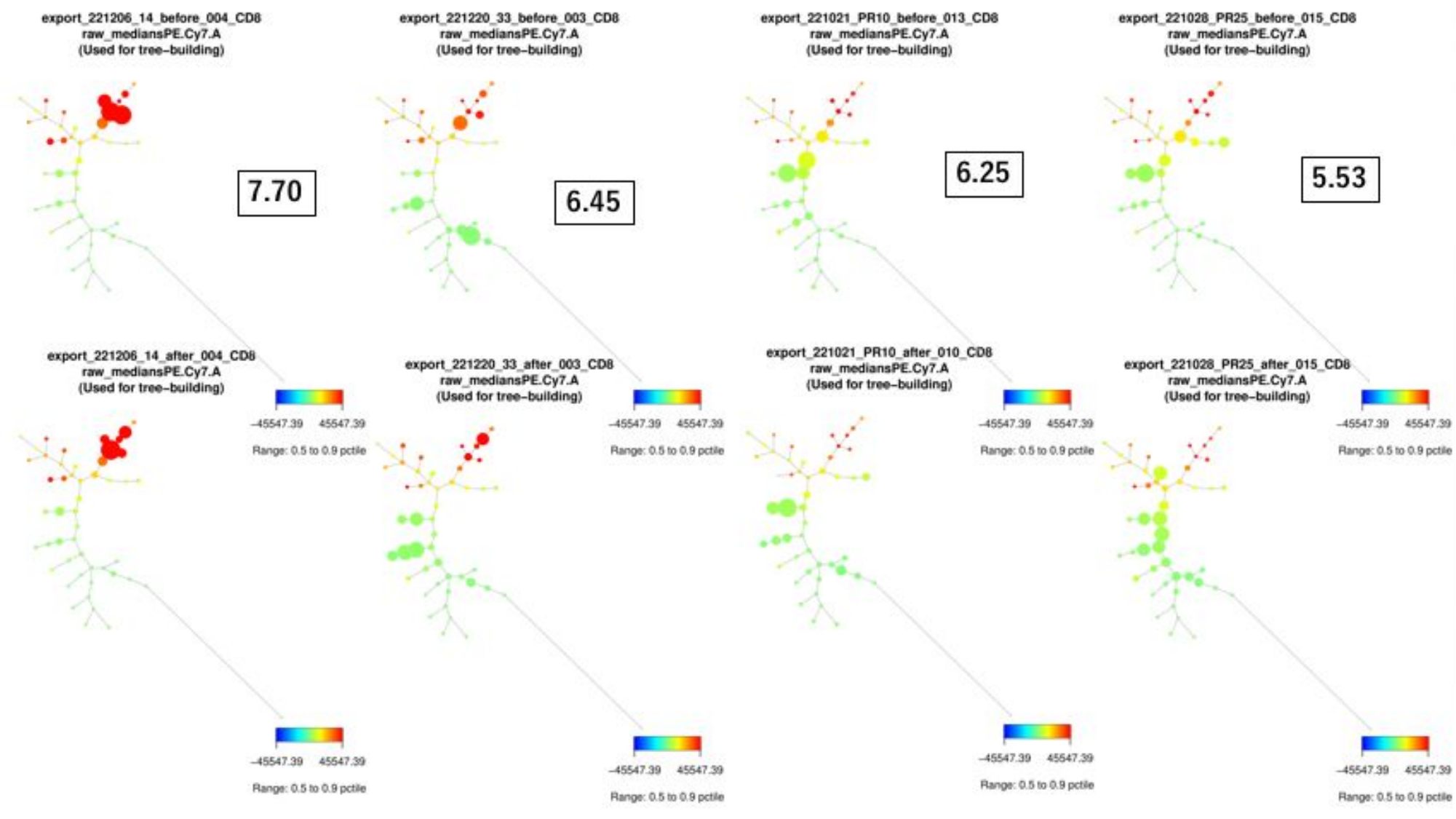

#

## Slide 8
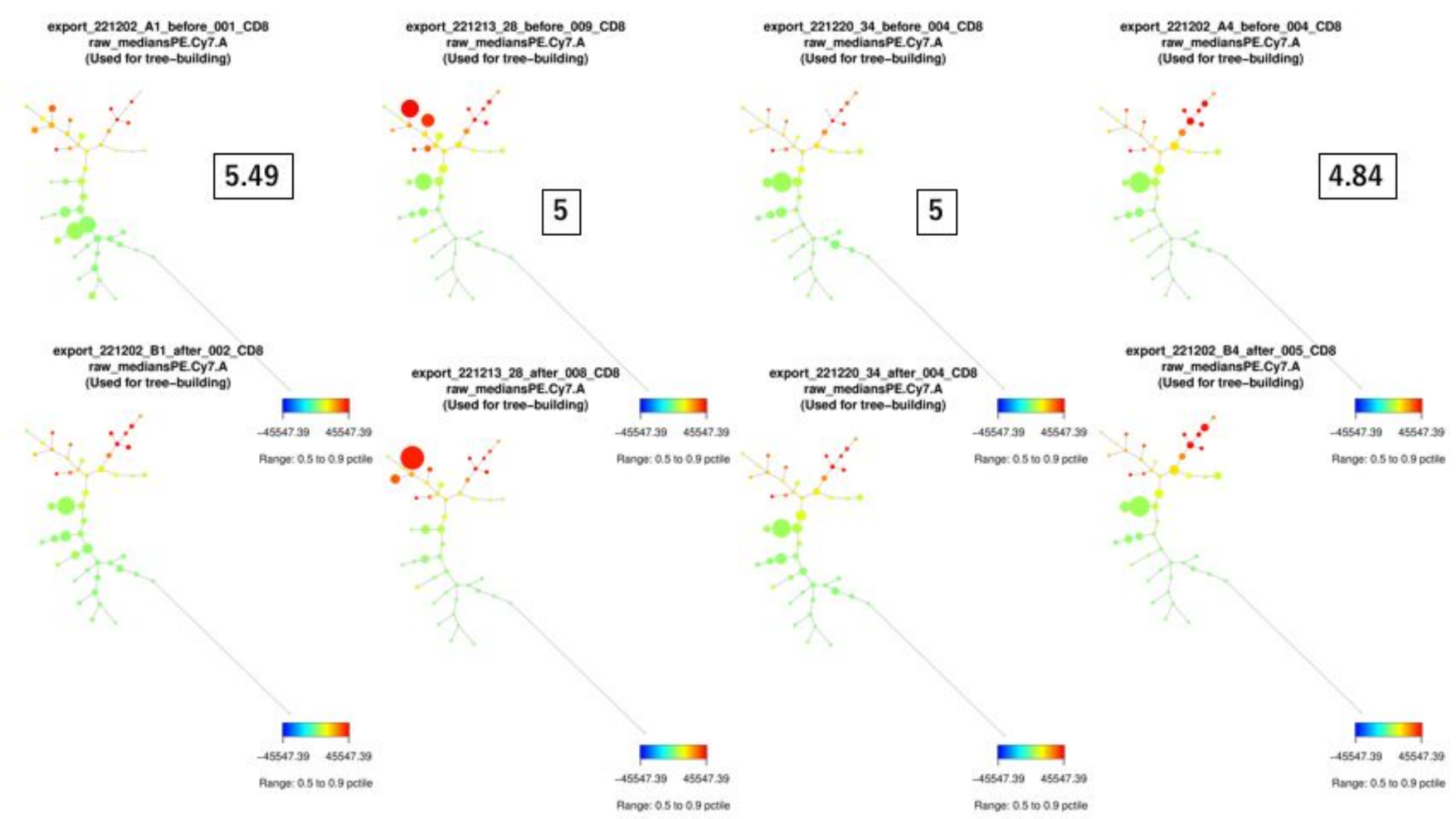

#

## Slide 9
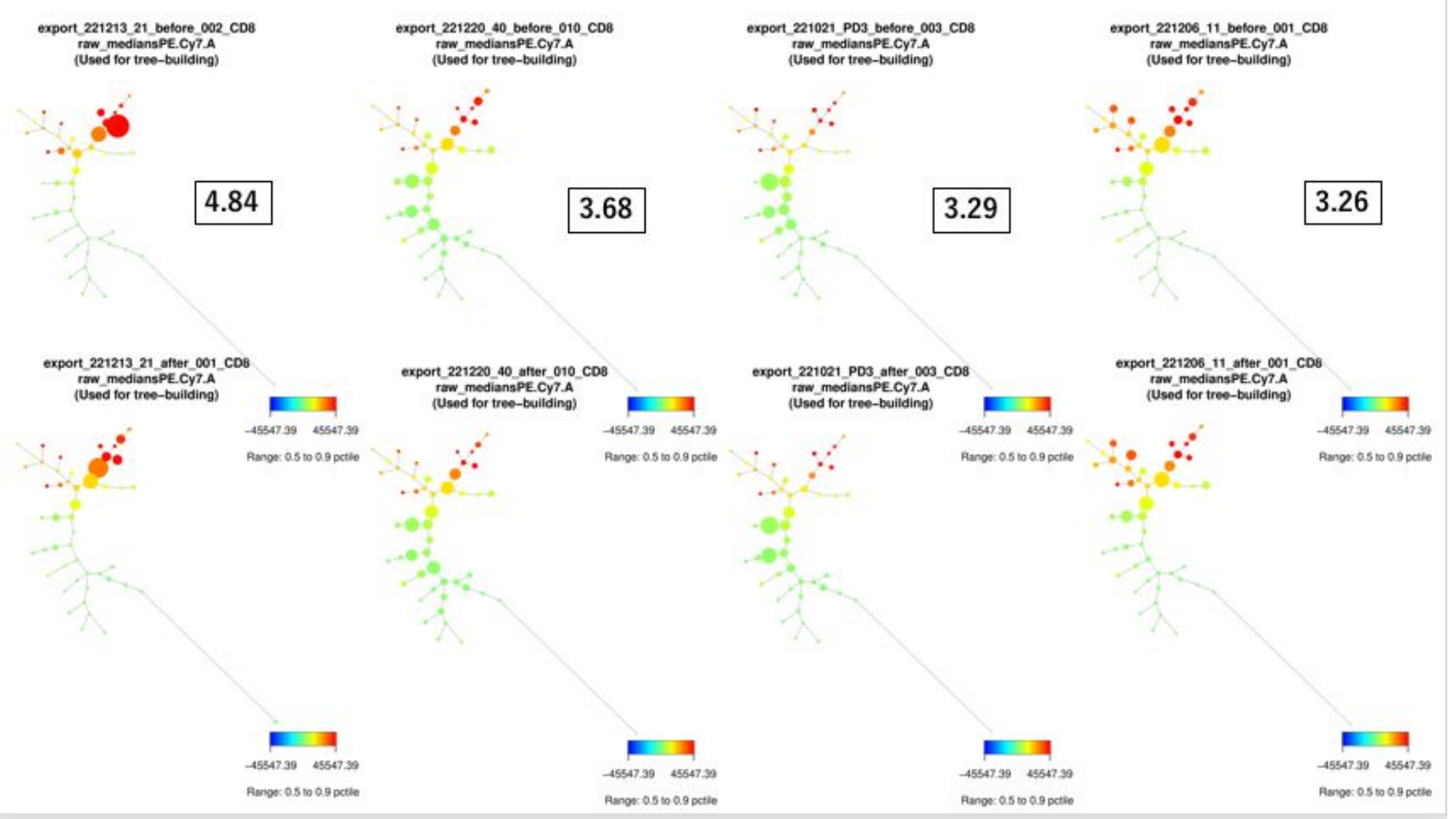

#

## Slide 10
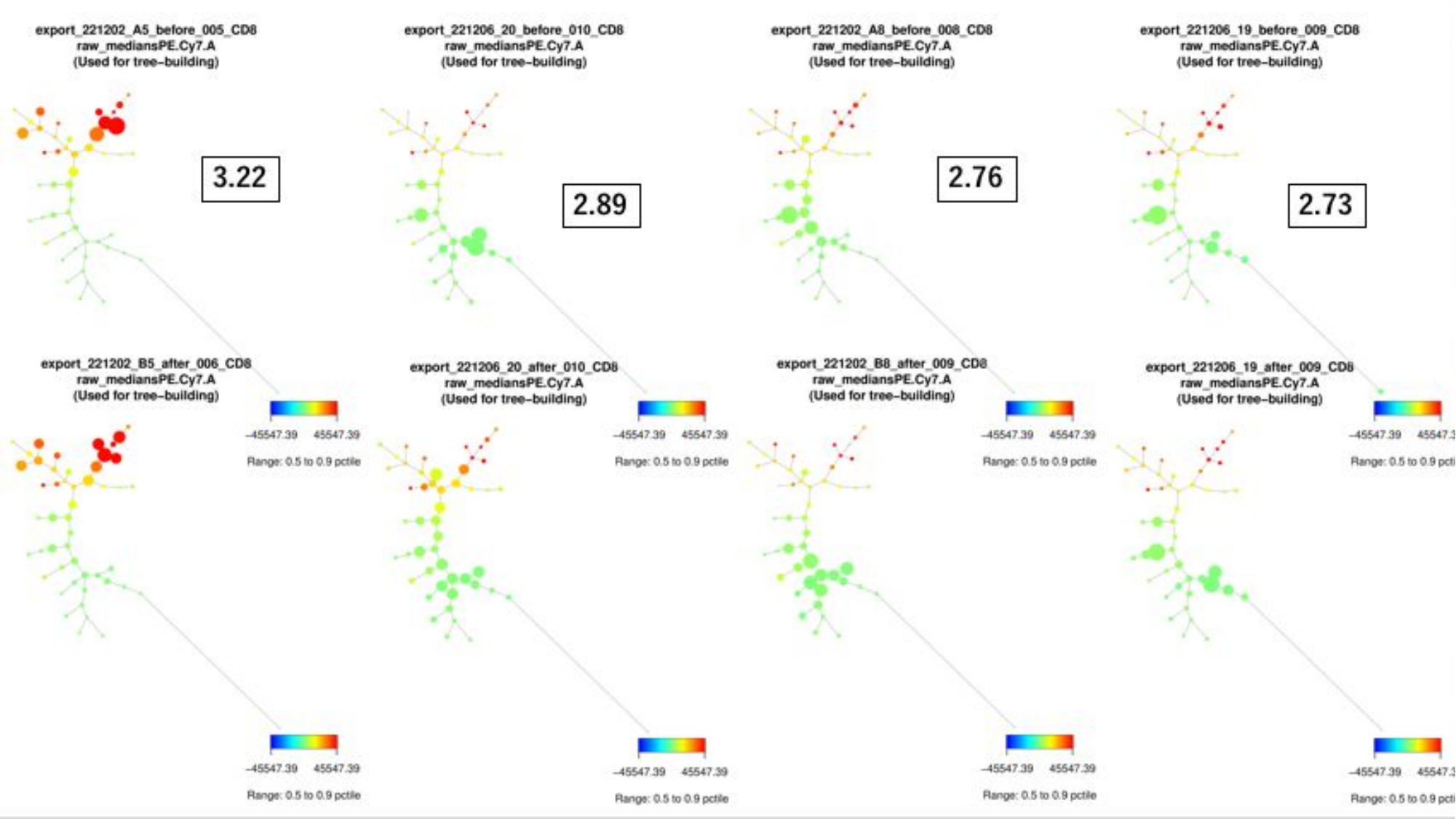

#

## Slide 11
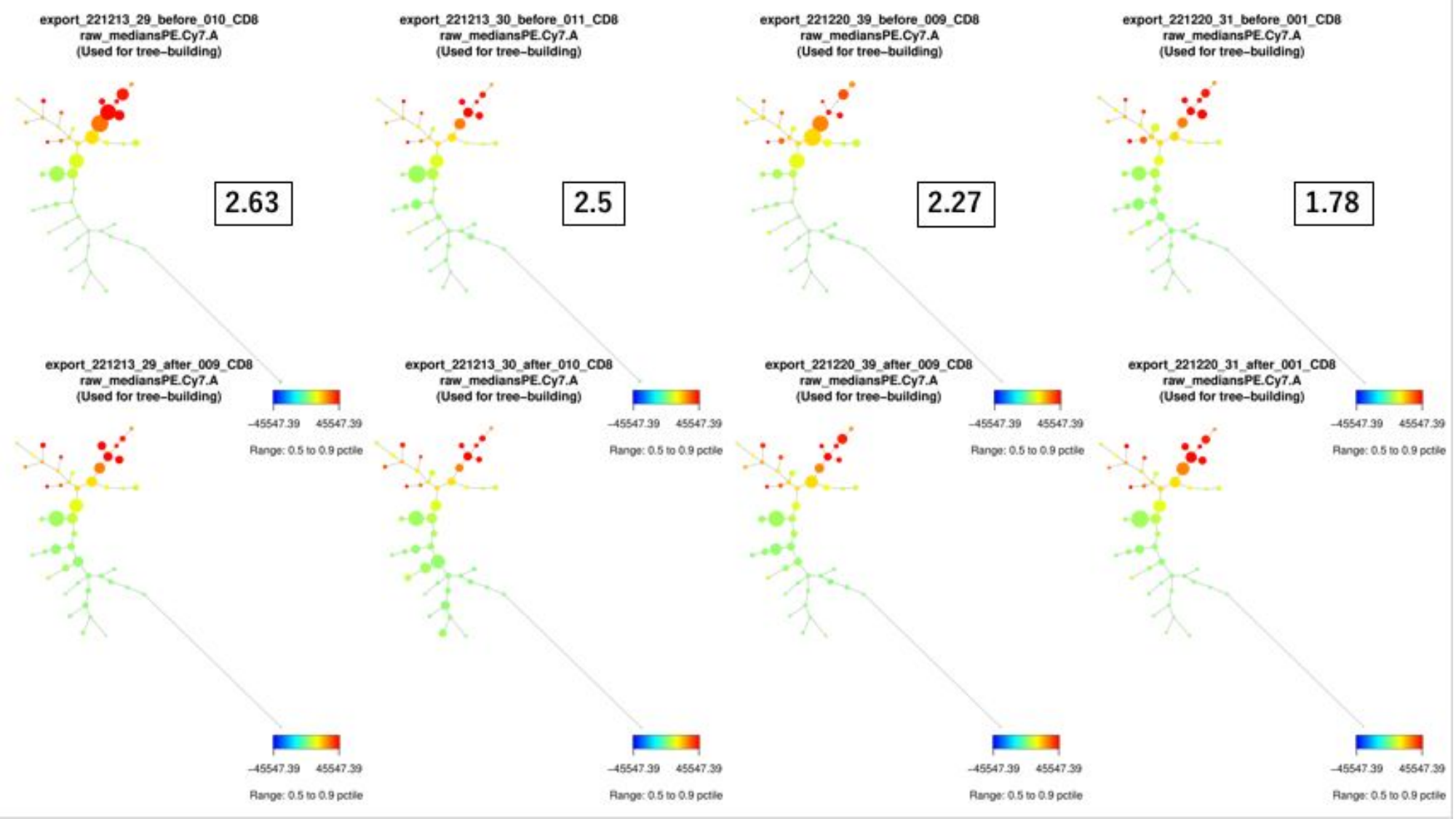

#

## Slide 12
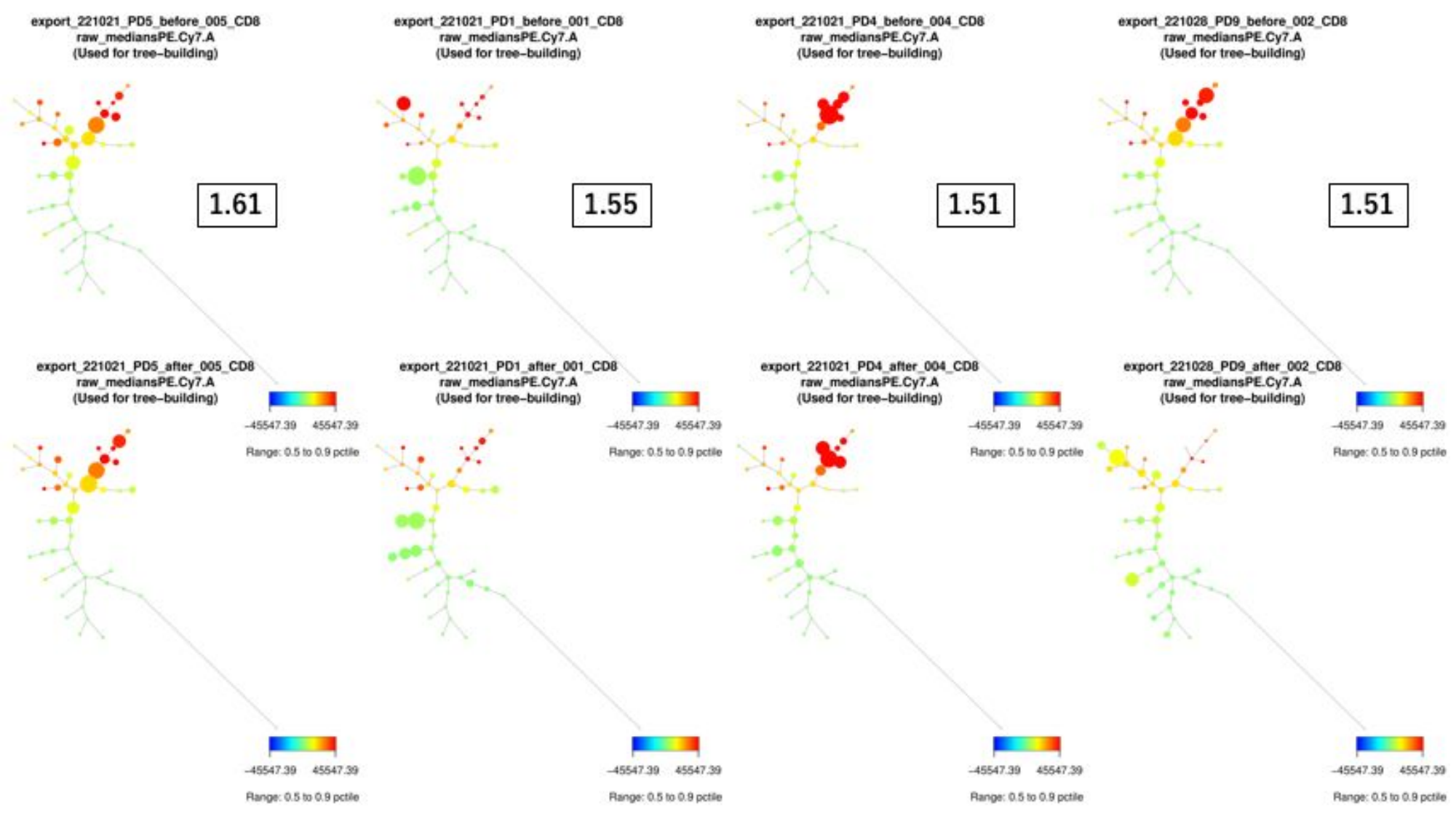

#

## Slide 13
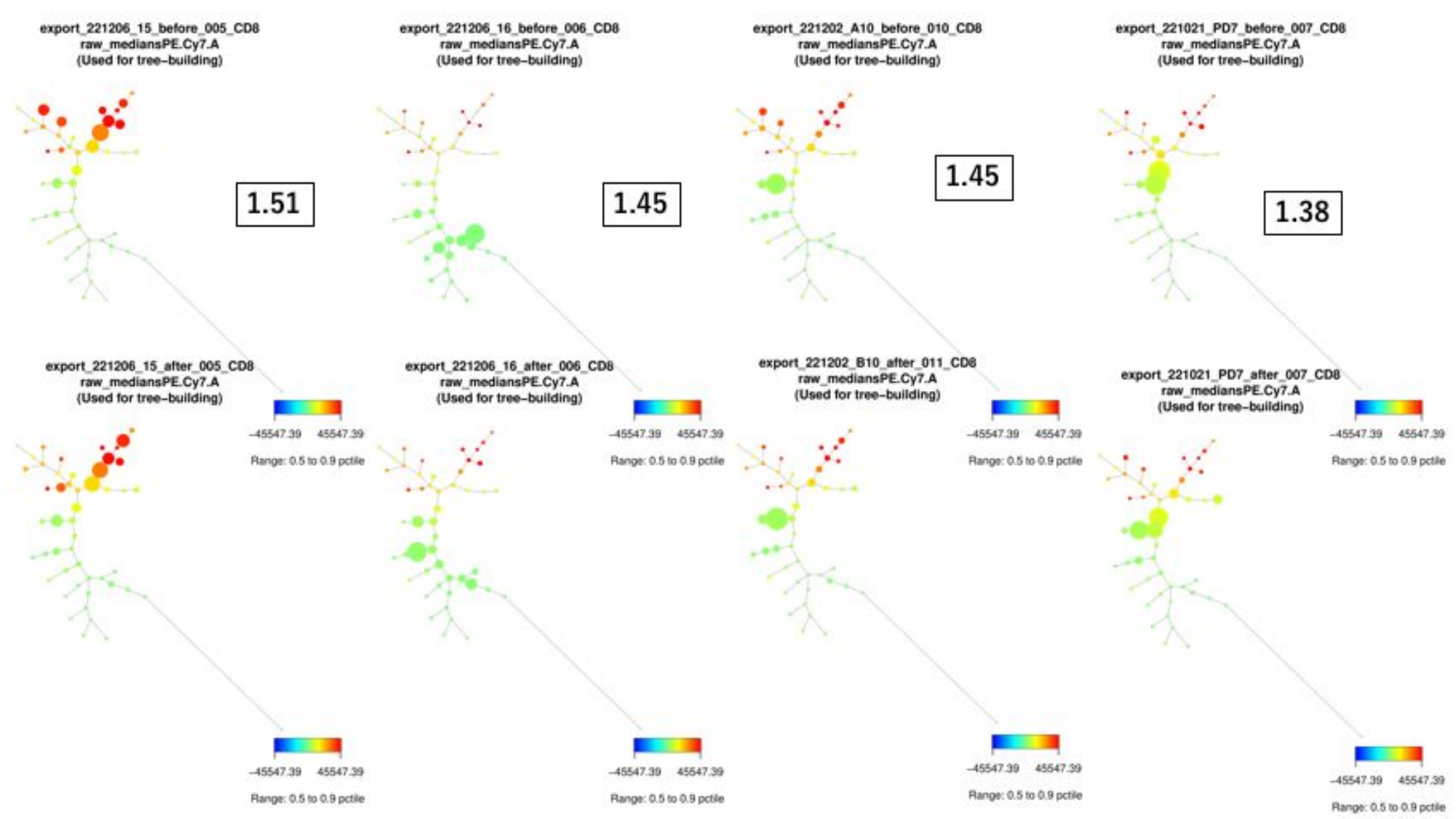

#

## Slide 14
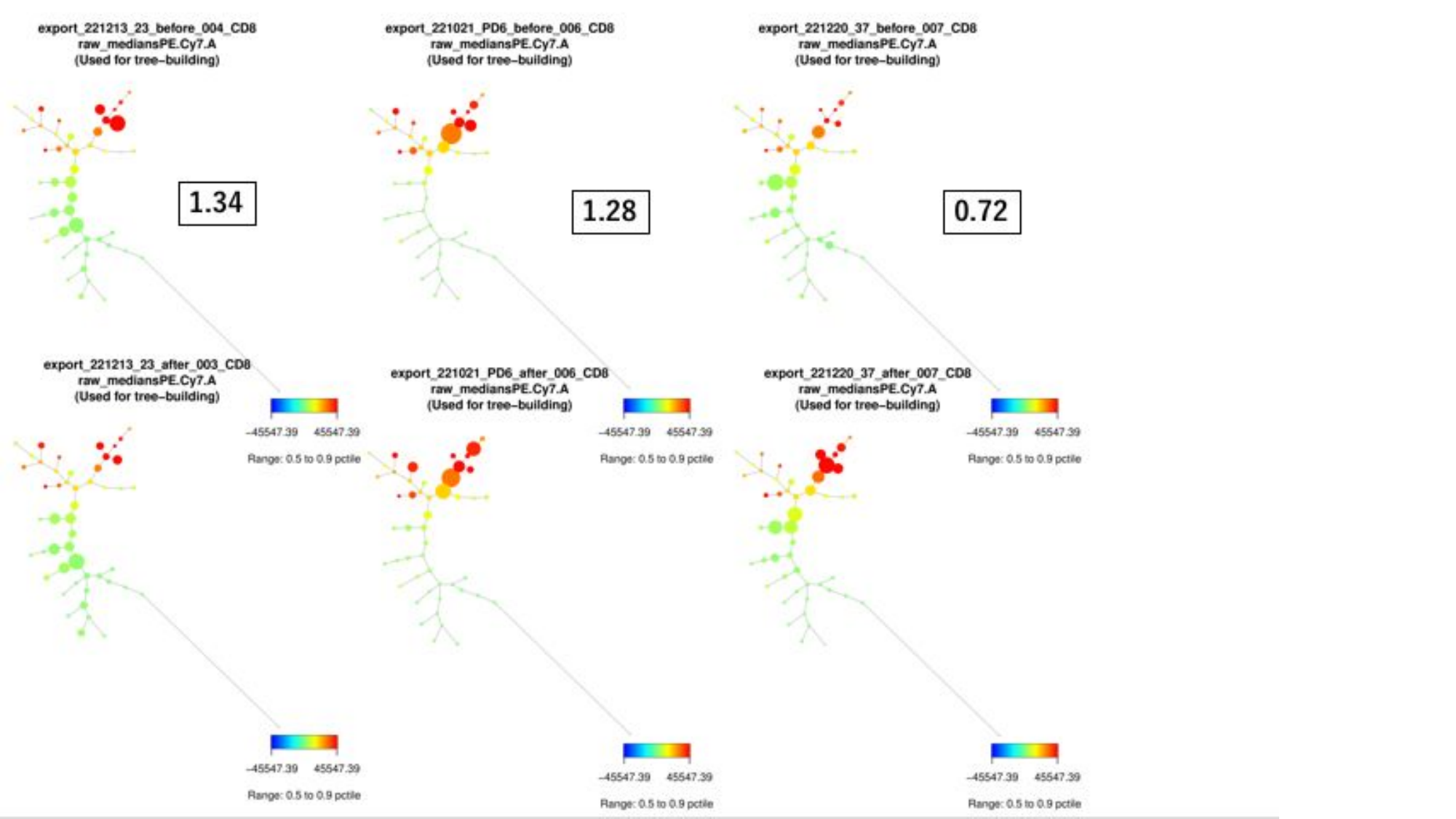

Supplement: Supplementary file 1 [file cancers-16-01328-s001.zip › Supplementary Figure S3.pptx]

## Slide 1
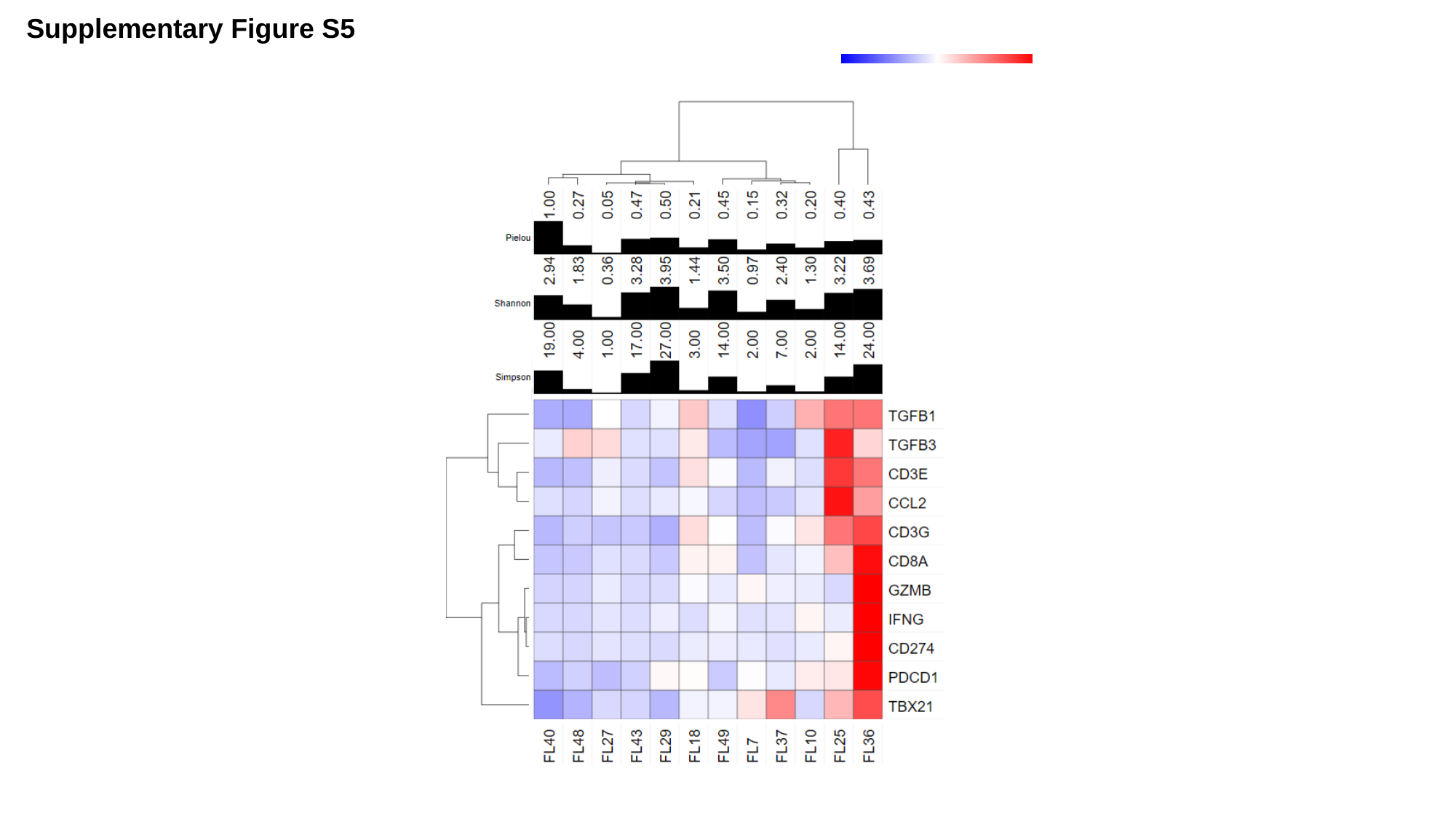

Supplementary Figure S5

Supplement: Supplementary file 1 [file cancers-16-01328-s001.zip › Supplementary Figure S5.pptx]
